# Global transcriptional and circadian regulation in a halotolerant cyanobacterium *Halothece* sp. PCC7418

**DOI:** 10.1038/s41598-022-17406-6

**Published:** 2022-08-12

**Authors:** Rungaroon Waditee-Sirisattha, Hiroshi Ito, Hakuto Kageyama

**Affiliations:** 1 Department of Microbiology, Faculty of Science, Chulalongkorn University, Phayathai Road, Pathumwan, Bangkok, 10330 Thailand; 2 Faculty of Design, Kyushu University, Fukuoka, 815-8540 Japan; 3 Department of Chemistry, Faculty of Science and Technology, Meijo University, 1-501 Shiogamaguchi, Tenpaku-ku, Nagoya, Aichi 468-8502 Japan; 4 Graduate School of Environmental and Human Sciences, Meijo University, 1-501 Shiogamaguchi, Tenpaku-ku, Nagoya, Aichi 468-8502 Japan

**Keywords:** Microbiology, Molecular biology

## Abstract

Substantial evidence has been accumulated about the molecular basis underlying halotolerance; however, insights into the regulatory networks for relevant genes and mechanisms of their interplay remain elusive. Here, we present a comprehensive transcriptome investigation, using RNA sequencing, of specific metabolic pathways and networks in a halotolerant cyanobacterium, *Halothece* sp. PCC7418, including the circadian rhythm profile. Dissecting the transcriptome presented the intracellular regulation of gene expressions, which was linked with ion homeostasis, protein homeostasis, biosynthesis of compatible solutes, and signal transduction, for adaptations to high-salinity environments. The efficient production and distribution of energy were also implicated in this acclimation process. Furthermore, we found that high-salinity environments had a dramatic effect on the global transcriptional expression regulated by the circadian clock. Our findings can provide a comprehensive transcriptome for elucidating the molecular mechanisms underlying halotolerance in cyanobacteria.

## Introduction

Cyanobacteria are widely distributed in nature, with some species capable of growing in unusual or extreme environments. *Halothece* sp. PCC7418 (originally identified as *Aphanothece halophytica*^1^; hereafter referred to as *Halothece*), isolated from the Dead Sea, is classified as a halotolerant cyanobacterium and shows a remarkable salt tolerance up to 3 M NaCl^2^. The biosynthesis and accumulation of the compatible solute glycine betaine (GB) is one of the molecular mechanisms that mediates a high degree of salt tolerance. The biosynthetic pathway of GB is already known; it is synthesized via a three-step methylation reaction using glycine as a precursor^3^. GB adapts an organism’s internal osmotic pressure to the extracellular environment. Mycosporine-2-glycine (M2G), a multifunctional compound, has also recently been suggested to function as a compatible solute^4^; however, the intracellular content of M2G in *Halothece* is extremely low compared to GB (It can also be confirmed in Fig. S9 of this paper), so it might be of low physiological importance as a compatible solute. Strategies to prevent the influx of excess sodium ions into the cell are also important. It has been shown that the Na^+^/H^+^ antiporter and the ATP-driven Na^+^-pump (Na^+^-ATPase) are involved in salt-tolerance mechanisms^2,5,6^. Despite several important strategies for salt tolerance having been clarified, the comprehensive intracellular regulation and interlinked pathways that trigger salt-tolerance mechanisms are still being uncovered. For example, how is the energy homeostasis (consumption vs compensation) maintained, a fundamental requirement for adaptation to high-salt levels? How is glycine provided, which is required for the production of large quantities of GB and M2G? Does salt stress affect circadian timekeeping mechanisms?

Considerable progress has been made in our understanding of the molecular, cellular, and physiological mechanisms involved in salt tolerance in *Halothece*. The subsistence of cyanobacteria is directly dependent upon light, therefore a comprehensive understanding of metabolism in cyanobacteria requires the effects of light–dark cycles and circadian regulation to be taken into account. A further important challenge is how the circadian clock molecularly implements salt-specific responses. Here, we conducted an intensive comparison of genome-wide transcriptional expression patterns in *Halothece* adapted to low- and high-salt concentrations under light–dark cycles prior to switching to continuous light. We then proposed a molecular network model for acclimation to high-salinity environments. In this model, energy production; protein turnover; primary and secondary metabolic pathways; ion efflux and influx; and signaling pathways, including the circadian clock, are interlinked to trigger salt-tolerance mechanisms and enable cells to withstand high-salt concentrations.

## Results and discussion

### Global transcriptional landscape in salt-acclimated *Halothece*

**Figure 1:**
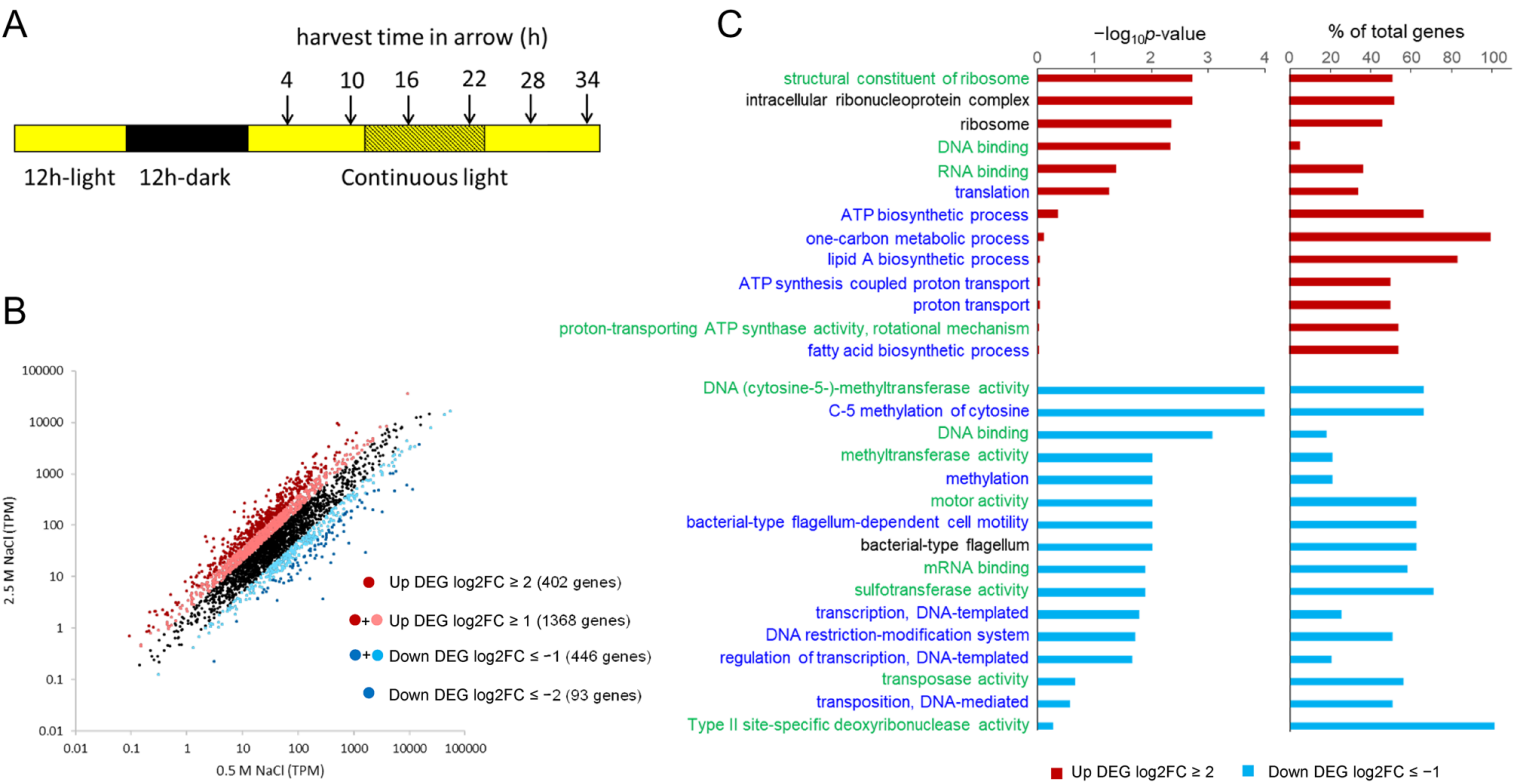
The gene expression pattern in high-salt-acclimated *Halothece* and GO analysis of DEGs. (**A**) Light–dark regimens and harvesting times. The hatched box indicates subjective night. (**B**) Scatter plot of RNA-seq data for low salt-acclimated cells vs. high-salt-acclimated cells. The details are given in the text. (**C**) GO analysis. There were 402 highly upregulated (log2FC ≥ 2) and 446 downregulated (log2FC ≤  − 1) genes analyzed. GO terms with *p* values ≤ 0.1 were selected, and GO terms with 0.1 < *p* value < 0.5 and with the percentage of DEGs out of the total number of genes exceeding 50% were listed. Blue, green, and black letters denote the Gene Ontology subcategories of biological processes, molecular functions, and cellular components, respectively.

To compare global transcriptional regulation of *Halothece* growing under high (2.5 M) and low (0.5 M) NaCl conditions, we conducted RNA-sequencing (RNA-seq) analysis. To identify differentially expressed genes (DEGs) under high-salinity conditions, we used the average transcripts per million (TPM) values at four time-points under continuous light (LL) conditions, to control for changes in transcript levels that are due to endogenous circadian rhythms. Two independent experiments were performed (Fig. S1A and S1B), and the average values were used for comparison. The sampling regime is illustrated in Fig. 1A. The growth rates of these cultures were shown in Figure S1C and S1D. Using the criterion of log2|fold change| (log2|FC|) ≥ 1, 1368 upregulated genes and 446 downregulated genes were identified from a total of 3843 genes (Fig. 1B and Table S1). When the criterion of log2|FC|≥ 2 was used, 402 highly upregulated and 93 highly downregulated genes were extracted. In comparison, in salt-acclimated moderately halotolerant cyanobacterium *Synechocystis* sp. PCC6803, 147 upregulated and 228 downregulated genes were extracted from 3079 genes, using a DNA microarray, at a threshold value of log2|FC|≥ 1^7^ (Table S1). In another example, RNA-seq analysis using salt-acclimated *Prochlorococcus* found 31 upregulated genes and 38 down-regulated genes from a total of 1959 genes, using the criterion log2|FC|≥ 2^8^ (Table S1). Thus, *Halothece* exhibited a large proportion of upregulated genes, suggesting this organism has a unique salt-tolerance mechanism. The list of all up- and down-regulated genes is shown in Dataset S1.

DEGs were submitted for refinement and functional clustering analysis based on Gene Ontology (GO). The functional categories of DEGs revealed that the 402 highly expressed genes (log2FC ≥ 2) included factors involved in ribosomes (GO:0,003,735, 0,005,840), nucleic acid binding (GO:0,030,529, 0,003,677, 0,003,723), and translation (GO:0,006,412) (Fig. 1C). Regarding ribosomes, DNA microarray analysis has shown that salt stress increases the expression of some genes for ribosomal proteins (*rpl2*, *rpl3*, and *rpl4*) in *Synechocystis* sp. PCC6803^7^. In addition, there was a large percentage of upregulated genes belonging to categories that included ATP production (GO:0,006,754, 0,015,986, 0,046,933), one-carbon metabolic pathway (GO:0,006,730), proton transport (GO:0,015,986, 0,015,992, 0,046,933), and lipid biosynthesis (GO:0,009,245, 0,006,633). On the other hand, the 446 genes whose expression was repressed (log2FC ≤  − 1) included diverse categories, such as methylation (GO:0,003,886, 0,090,116, 0,008,168, 0,032,259), nucleic acid binding (GO:0,003,677, 0,003,729), molecular motors (GO:0,003,774, 0,071,973, 0,009,288), sulfotransferase (GO:0,008,146), DNA transposition (GO:0,004,803, 0,006,313), transcription (GO:0,006,351, 0,006,355), and nuclease (GO:0,009,307, 0,009,036) (Fig. 1C).

## Insights into the transcriptional regulation of specific pathways in high-salt-acclimated *Halothece*

### A robust system for restriction of Na^+^ accumulation determines halotolerancy in *Halothece*

Excess Na^+^ accumulation is the key ion toxicity under salt stress. As a halophilic cyanobacterium and thus native to a hypersaline environment, *Halothece* possesses a robust mechanism that ensures its survival in extreme salt concentrations. The *Halothece* genome encodes a number of predicted Na^+^ and H^+^ pumping proteins (Na^+^-ATPase and H^+^-ATPase), cation/proton antiporters (CPA1-3), and NhaC family antiporters. These membrane proteins are presumably responsible for imparting salt tolerance through the transport dynamics of ion homeostasis. CPA1 and CPA2 function to catalyze the efflux of cytoplasmic toxic ions, such as Na^+^ and Li^+^ or excess K^+^, in exchange for protons^9^. Ten genes are annotated as CPA1 and CPA2 superfamilies in *Halothece*. As shown in Fig. S2 and Table S2, except for *H1519*, all of these genes were upregulated in salt-acclimated cells. In particular, *H0995*, *H0978*, *H3396*, and *H3842* were upregulated more than 2.0 of log2FC. Previous investigation revealed that H0978 (Ap-NapA1-2) and H3396 (Ap-NapA1-1) are essentially Na^+^/H^+^ antiporters, serving as key elements for tolerance to Na^+^ toxicity and adaptation to an alkaline pH^5^. H0978 and H3396 exhibited high sequence similarities (57% and 66% identities, respectively) with an essential Na^+^/H^+^ antiporter, NhaS3 (Sll0689), found in *Synechocystis* sp. PCC6803. NhaS3 is thought to be associated with salt tolerance in *Synechocystis* sp*.* PCC6803^10^. The upregulation of the multi-cistronic operon CPA3, which is a member of the Na^+^ transporting Mrp (Multiple resistance and pH) superfamily, was also observed (Fig. S2 and Table S2). The proteins encoded in the gene cluster consisting of *H0970*-*H0977* were previously characterized as constituents of an active CPA3 antiporter, Ap-Mrp. Owing to its ability to exhibit Na^+^/H^+^ exchange activity, it has been suggested that Ap-Mrp is involved in salt stress tolerance^6^.

A robust ion transport system in *Halothece* was further supported by the presence of a distinct antiporter family, NhaC, which has so far been found exclusively in alkalinophiles, such as the genus *Bacillus* and some pathogenic bacterial species^9^. *H2569*, which encodes an NhaC-type antiporter, was upregulated (Fig. S2 and Table S2). An analysis of gene co-occurrence across cyanobacterial genomes revealed that NhaC is present in a few cyanobacterial species (*Halothece* sp. PCC7418 (this study), *Gloeocapsa* sp. PCC7428*, Rivularia* sp. PCC7116*,* and *Leptolyngbya* sp. PCC7375). Among these strains, *Halothece* and *Rivularia* are halophilic. RT-PCR analysis showed that this gene is highly upregulated under salt and alkaline stresses (Fig. S3). NhaC was initially identified from *Bacillus* and demonstrated to show membrane-associated Na^+^/H^+^ exchange activity^11^. The physiological role of NhaC in *Bacillus* and pathogenic bacteria, such as *Staphylococcus aureus*, is associated with Na^+^ and K^+^/H^+^ antiport, as well as maintaining cytoplasmic pH during alkaline stress^9^.

CPAs rely on a proton gradient to expel ions from cells, reducing toxicity. The membrane protein H^+^-ATPase is known to be responsible for the establishment of a proton gradient. H^+^-ATPase is encoded by a multigene family. *Halothece* possesses the typical thylakoid-located ATP-driven H^+^-pump (H^+^-ATPase), comprising H0416–H0423 and H1964–H1965. The enzyme is considered to produce ATP by photophosphorylation in which ATP synthesis proceeds using the proton gradient generated as a result of electron transfer associated with photosynthesis (Fig. S2). As shown in Fig. S2 and Table S2, all constituent genes were strongly upregulated, suggesting a crucial role for the formation of a proton motive force and powering the formation of ATP under high-salinity conditions. The involvement of CPA and the H^+^-ATPase is thus tightly bound. The elevated expression of the major subunits of NAD(P)H quinone oxidoreductase, cytochrome c oxidase, and cytochrome *b*6*f*. complex (*petA* and *petB*), which are involved in proton accumulation in the thylakoid lumen, also supports that ATPase actively produces ATP under salt-stress conditions (Fig. S2 and Table S2).

An additional system for maintaining Na^+^ homeostasis in cyanobacteria was suggested by the function of the ATP-driven Na^+^-pump (Na^+^-ATPase; Na^+^ pump), by coupling the hydrolysis of ATP to the translocation of Na^+^ ions across the cell membrane^2^. In *Halothece*, the Na^+^-ATPase operon comprising *H2520*–*H2528* was demonstrated to participate in salt-tolerance mechanisms^2^. The expression of *H2520*–*H2524* was upregulated while the expression of *H2525*–*H2528* was downregulated in high-salt-acclimated *Halothece* cells (Fig. S2 and Table S2). Differences in transcriptional expression patterns in the anterior and posterior regions indicated that these nine genes might be transcriptionally regulated by multiple promoters. *H2520* encodes a gamma subunit. It has been shown that the gamma subunit in Na^+^-ATPase plays a modulatory role upon association with functional membrane partners.

Taken together, the varied pattern of transcriptional responses of the CPA superfamilies, NhaC, H^+^-ATPase and Na^+^-ATPase, strongly suggested that *Halothece* could preferentially rely on proton motive force (H^+^ gradient) or sodium motive force (Na^+^ gradient) as sources of energy during salt stress. Moreover, these primary active transport mechanisms mediate several Na^+^/H^+^ antiporters in CPA1-3, including the NhaC family. These antiporters could be mutual elements that help to provide a robust mechanism for salt tolerance.

### Protein homeostasis

As confirmed by GO analysis (Fig. 1C), translation-related factors such as ribosomal proteins and elongation factors tended to be extremely highly expressed in *Halothece* cells adapted to a high-salt environment (Fig. S2 and Table S2). As this tendency is also observed in other cyanobacterial strains^7,8^, accelerated protein synthesis is considered to be a common salt-tolerance mechanism. It should be noted that the protein synthesis process consumes a large quantity of ATP^12^. On the other hand, there was also a trend of increased expression among protease family genes. *Halothece* has a large number of serine proteases compared with other cyanobacteria^13^, and 15 of 30 of these genes were significantly upregulated (Fig. S2 and Table S2). The upregulated genes included ATP-dependent Clp and ATP-independent HtrA proteases (Table S2). In addition, four of the five genes that encode FtsH, which is ATP-dependent metalloproteinase, showed markedly increased expression (Fig. S4 and Table S2). As reactive oxygen species (ROS) produced under salt stress can damage intracellular proteins^14^, it is thought that protein turnover is activated to remove denatured proteins. FtsH, which belongs to the AAA protein family, has been reported to play a vital role in the removal of misfolded proteins in favor of cell homeostasis^15^. As shown in Table S3, the total amount of soluble protein extracted from *Halothece* was lower under 2.5 M NaCl conditions than under 0.5 M NaCl conditions. This observation might suggest that there was upregulation of the protein degradation system although protein turnover and intracellular protein contents are not directly linked. As protein turnover involves the consumption of energy, the efficient production of ATP is essential.

### Specific transcriptional responses via compatible solutes and one-carbon metabolic pathway

*Halothece* is a well-established microbial host of GB accumulation^3,16^. As the major compatible solute in this cyanobacterium, GB is used to make considerable adjustments in intracellular osmotic pressure in response to the surrounding high-salinity environment. In our study, RNA-seq analysis revealed the regulatory picture of transport and biosynthesis of GB. For GB transport, three ABC (ATP-binding cassette) transporters and a BCCT (betaine/carnitine/choline transporter) were found, and their gene expression tended to increase in high-salt-acclimated *Halothece* (Fig. S4 and Table S2). In particular, the expression levels of *H3058* and *H3406* were markedly increased, which is considered to contribute to the uptake of external GB. For de novo bioproduction of GB, sufficient glycine and methyl donors are required, because GB is biosynthesized via a three-step methylation reaction using glycine as a precursor^3^. The major supply pathway of glycine and methyl donors is the one-carbon metabolic pathway that includes folate and methionine metabolism. As shown in Fig. S5 and Table S2, the gene expression of enzymes involved in these pathways was upregulated in salt-stress-acclimated cells. The increased gene expression of serine hydroxymethyltransferase (SHMT) H1525, an enzyme that reversibly interconverts glycine and serine, resulted in a log2FC value of 2.7. The light-independent phosphoserine pathway and light-dependent photorespiratory pathway together with SHMT are known to be involved in glycine and serine interconversion (Fig. S6). In the photorespiratory pathway, the expression of glutamate:glyoxylate aminotransferase (GGAT) H3470, which is responsible for the conversion of glyoxylate to glycine, was significantly upregulated (Fig. S6 and Table S2). On the other hand, H3470 is also known to function as a phosphoserine aminotransferase (PSAT), in the phosphoserine pathway^17^. In this pathway, we also observed the marked upregulation of 3-phosphoglycerate dehydrogenase (PGDH, H2641) and phosphoserine phosphatase (PSP, H0286), which convert 3-PG to serine. Most of the enzymes that catalyze the Calvin–Benson cycle were also markedly induced (Fig. S6 and Table S2). For the three-step methylation of GB, *S*-adenosylmethionine (SAM) is considered to be the main methyl donor in this pathway. Three genes involved in one-carbon metabolism encode methionine adenosyltransferase (or AdoMet synthase, H2062), 5,10-methylenetetrahydrofolate reductase (MTHFR, H0548), and aminomethyltransferase (AMT, H0371). All of these proteins are crucial for integrating carbon unit as well as providing substrates for methylation reactions. Notably, two genes in the *Halothece* genome were annotated as AdoMet synthase (*H2062* and *H3028*). H2062 showed high sequence similarity (95% identity) with an AdoMet synthase (WP_146294713) of the halophile *Euhalothece natronophila*, while H3028 showed sequence similarity (71% identity) with AdoMet synthase (WP_067769922.1) of the filamentous cyanobacterium *Nostoc* sp. NIES-3756. These results suggest that the one-carbon metabolic pathway, the carbon-fixation pathway, and the serine synthesis pathway are tightly orchestrated in *Halothece* under salt-stress conditions, resulting in the massively increased synthesis of GB.

The enhancement of glycine production may also be related to the biosynthesis of mycosporine-like amino acids (MAAs). MAAs are generally known as ultraviolet (UV)-absorbing compounds but may also play a role as osmotic compatible solutes^18^. More than 50 different structures of MAAs have been reported so far, with the most common structure being one or two amino acids attached to the core structure. It is known that mycosporine-2-glycine (M2G), which consists of two glycines, is biosynthesized in *Halothece*^4^. The expression of 3-dehydroquinate synthase (*DHQS, H1590*), *O*-methyltransferase (*O-MT, H1078*), and ATP-grasp protein (*ATP-grasp, H1077*) among the four M2G synthesis genes was increased in high-salt-acclimated cells, and the expression level of *H1078* was log2FC > 1 (Table S2).

### Expression profiling of a two-component system delineates the signaling network

Two-component systems (TCSs) are among the most prevalent signaling systems that mediate environmental stress responses. A TCS is typically composed of a sensory histidine kinase (HK) and the corresponding response regulator (RR). The *Halothece* genome comprises a significant enrichment of genes encoding TCS, suggesting its genome is highly adaptable to changing environmental conditions. Our genome-based analysis revealed a total of 90 TCS genes (Table S2). The distribution of these TCS genes showed that they were encoded as 72 orphans, 7 pairs, 1 triad (HK-RR-HK), and 1 tetrad (HK-RR-RR-HK). It should be noted that hybrid histidine kinases (HyKs), which contain both HK and RR domains, are classified as HKs. In high-salt-acclimated *Halothece* cells, eight HKs and ten RRs were found to be upregulated (log2FC > 1) (Fig. S7A and Table S2. These factors may be associated with high-salt acclimation. It was previously reported that for *Synechocystis* sp. PCC6803, Hik2 (Slr1147), Hik10 (Slr0533), Hik16 (Slr1805), Hik33 (Sll0698), Hik34 (Slr1285), and Hik41 (Sll1229) are involved in the salt stress response^19,20^. Among them, in *Halothece*, H2035 and H3509 showed similarities to Hik33 (64% identity) and Hik34 (69% identity), respectively, but the expression of both was repressed (Fig. S7A and Table S2). On the other hand, the RR H0084, which showed similarity (85% identity) to Rre31 (Slr0115), identified as a partner of Hik33^20^, was highly expressed. This RR, also known as RpaA (regulator of phycobilisome association A), has been suggested to play an important role in the transcriptional regulation of circadian rhythms in *Synechococcus elongatus* PCC7942 by forming a TCS with SasA^21^. H0715, which corresponds to SasA, is strongly upregulated in high-salt-acclimated *Halothece* (Table S2), suggesting that the SasA-RpaA TCS may be involved in the adaptation to high salinity as well as the circadian clock. H3128, which corresponds to another SasA partner, RpaB, was also upregulated, but the expression level did not reach log2FC = 1 (Table S2). Putative interaction networks among upregulated (log2FC > 1) HKs and RRs are illustrated in Fig. S2B. This prediction suggests that the presence of multiple HK partners for a single RR results in complex transcriptional regulation for the acclimation to high salinity. As described above, we here focused on changes in transcripts that encode the HKs and RRs, but it should be noted that the direct factor for the transcriptional regulation of the target genes is the biochemical activation of HKs and RRs in response to the external environment. Biochemical analysis of HKs and RRs is indispensable for understanding the regulation of TCS during salt acclimation.

## Effects of high salinity on the circadian gene expression in high-salt-acclimated *Halothece*

### Alteration of circadian gene expression patterns in different salinity environments

**Figure 2:**
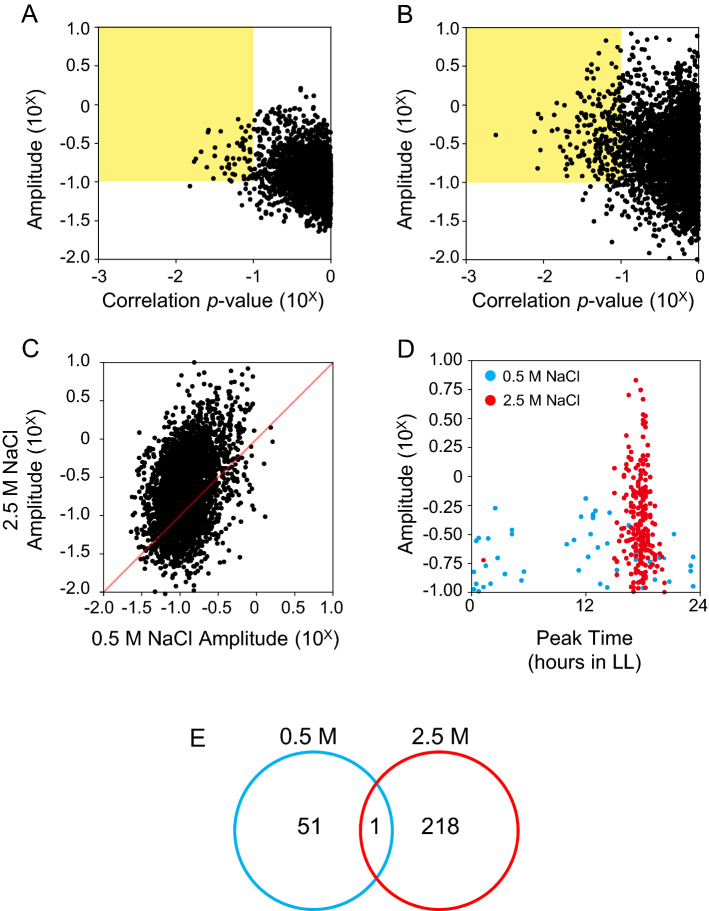
Genome-wide circadian transcription profiles in low- and high-salt-acclimated *Halothece* cells. (**A** and **B**) Distribution of the oscillatory indices; amplitude (ordinate) and correlation *p*-value (abscissa) from each transcript under constant light conditions in low- (**A**) and high- (**B**) salt-acclimated cells. The smaller values in amplitude and the larger values in correlation *p*-values from two experiments are displayed. We defined the genes in the yellow areas as the cycling genes. The definition of each index is described in the Materials and Methods. (**C**) Scatter plot for the amplitude of low- vs high-salt-acclimated cells. (**D**) Scatter plot for the peak expression time (ordinate) vs amplitude (abscissa) from low- and high-salt-acclimated cells. (**E**) Numbers of detected circadian cycling genes in low- and high-salt-acclimated cells.

**Figure 3:**
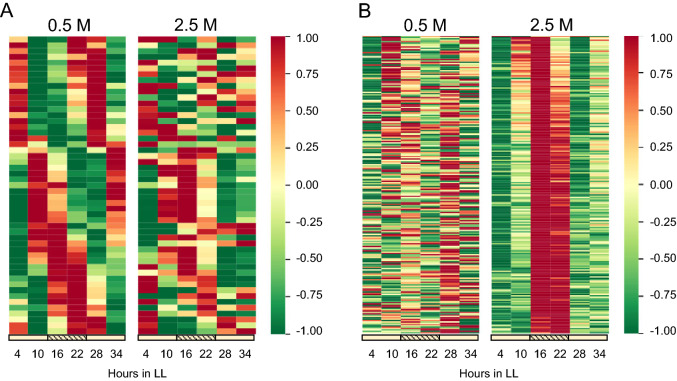
Circadian cycling expression profiles from low- and high-salt-acclimated cells. The average and SD of six time points (4, 10, 16, 22, 28 and 34 h in LL) are normalized to 0.0 and 1.0, respectively. The time course for each gene is displayed horizontally and the circadian cycling genes are placed vertically in ascending order of peak time of low- (**A**) and high-salt conditions (**B**).

To investigate the effects of salinity environment on the circadian clock, we analyzed the data collected in LL4–34 as described in the ‘Materials and Methods’ section and found that 51 and 218 genes exhibited circadian oscillation under 0.5 and 2.5 M NaCl conditions, respectively (Fig. 2A, B). The lists of cycling genes are shown in Dataset S2. Although there were more cycling genes with higher amplitude under 2.5 M NaCl conditions (Fig. 2C), most of the genes were in the same phase, with the peak at LL16-20 (Fig. 2D). On the other hand, the phases of cycling genes under 0.5 M NaCl conditions were widely distributed, between LL0–24 (Fig. 2D). Only one gene (*H0532*, aldehyde-alcohol dehydrogenase, *adhE*) was determined to be a cycling gene in both salt concentration conditions (Fig. 2E). Figure 3A depicts the phase-aligned heatmap of the genes that were rhythmically expressed in the 0.5 M NaCl condition and shows that the aligned patterns of the circadian phase were disrupted in the 2.5 M NaCl condition. A different expression pattern was also found when the cycling genes were aligned from the perspective of the cycling genes in the 2.5 M NaCl condition (Fig. 3B). These transcriptome profile heatmaps raised the possibility that salt stress resulted in the loss of rhythmic expression that was evident in 0.5 M NaCl. To the best of our knowledge, there have been no previous reports of such a large difference in the circadian expression patterns of genes under different salinity conditions. Among 51 cycling genes under 0.5 M NaCl condition, we could not find the genes previously shown to be involved in salt stress tolerance (Dataset S2). Regarding the 218 genes under 2.5 M NaCl conditions, although *H1519* (CPA1) and *H2522* (the delta subunit in Na^+^-ATPase), which were discussed above, were extracted as cycling genes, no other factors directly associated with the salt stress tolerance were found. These observations might indicate that the salt tolerance mechanism is not regulated by circadian clock in this cyanobacterium. However, it should be noted that in order to analyze more precise circadian rhythmicity and phase, it is necessary to perform sampling at more time points for a longer period of time.

**Figure 4:**
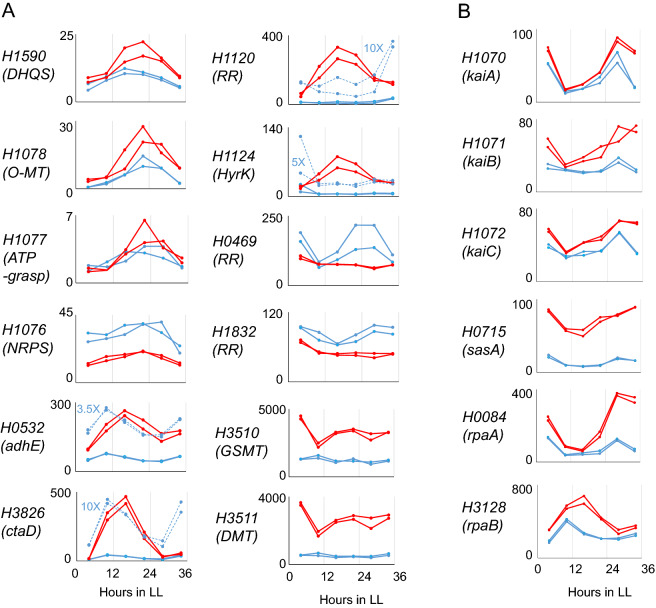
Temporal expression patterns of 22 representative *Halothece* genes. The blue and red lines indicate expression profiles under conditions of 0.5 and 2.5 M NaCl, respectively, from two independent experiments. The number on the ordinate indicates TPM values. (**A**) Genes that exhibited various expression patterns depending on NaCl concentration. (**B**) Circadian clock-related genes. To make it easier to see the expression patterns, the TPM values of *H0532*, *H3826*, *H1120*, and *H1124* are magnified by the magnification value indicated in each graph (dotted blue lines).

In Fig. 4A, several genes were picked up in terms of their relationship to the salinity condition and the circadian clock (the rhythmicity of gene expression described here includes those that were not determined to be “cycling” in Fig. 2A and B). The M2G synthesis genes, *H1590* and *H1078-1076*, showed similar peak times under both salinity conditions. *H0532*, which was extracted as a cycling gene under both salinity conditions, showed different phases of expression rhythms under different NaCl concentrations. Under 0.5 M NaCl, the peak appeared at LL10, but under 2.5 M NaCl, the peak was preferentially shifted to LL16. *H3826* (cytochrome c oxidase subunit I, *ctaD*) showed a similar pattern. On the other hand, *H1120* (RR) and *H1124* (HyK), which are related to TCS, showed no rhythm under 0.5 M NaCl conditions, while circadian expression with a peak at LL16 was observed under 2.5 M NaCl conditions. The RRs of TCS, *H0469* and *H1832*, oscillated under 0.5 M NaCl conditions but became arrhythmic under 2.5 M NaCl conditions. Two methyltransferases, *H3510* (glycine sarcosine methyltransferase, *gsmt*) and *H3511* (dimethylglycine methyltransferase, *dmt*), were examples of arrhythmic genes under both NaCl conditions. As shown in Figure S8A and B, intracellular amount of GB did not show the circadian oscillation under both NaCl conditions. For some of the genes discussed herein, RT-PCR analysis also confirmed similar phases and waveforms to the rhythms seen based on the RNA-seq data (Fig. S9).

### Relationship between the circadian clock in Halothece and the salinity of the environment

In Fig. 4B, the expression patterns of *kaiA*, *kaiB*, *kaiC*, *sasA*, *rpaA*, and *rpaB* are listed as factors related to the circadian clock. KaiA, KaiB, and KaiC are essential for the oscillation of the circadian rhythm in cyanobacteria^22^; SasA is a KaiC-binding protein that forms a TCS with RpaA to regulate global circadian transcriptional expression^21^; RpaB is also thought to be involved in circadian gene expression^23^. *kaiABC*, *sasA*, and *rpaA* showed rhythms peaking at around LL4 and LL28 under both NaCl concentrations, which was different from the average peak times for most genes under 2.5 M NaCl conditions.

Considering that the circadian expression of *kaiABC*, *sasA*, and *rpaA* showed the same peak time under both salinity conditions, it was thought that the phases of the central circadian oscillator did not change at different salt concentrations (Fig. 4B). Therefore, the existence of genes such as *H0532*, *H3826*, and *H3128*, whose peak times of expression rhythm changed with different salt concentrations, is strange (Fig. 4A and B). The LL16 peak in expression pattern shown by these genes in the 2.5 M NaCl conditions is consistent with most of the genes extracted as cycling genes in the 2.5 M NaCl concentration conditions (Fig. 3B). The presence of genes such as *H1120* and *H1124*, which were arrhythmic under 0.5 M NaCl conditions but showed a similar pattern of fluctuation under 2.5 M NaCl conditions, may also support that the cyclic pattern was induced not by the circadian clock but by the high-salinity conditions. In addition, the accumulation pattern of M2G under 0.5 M NaCl conditions showed a circadian oscillation, with an increase in accumulation during the subjective day, but the rhythm disappeared under 2.5 M NaCl conditions (Fig. S8C and D). The M2G biosynthetic genes (*H1590*, *H1078-1076*) showed fluctuations peaking at LL16-20 under both salinity conditions (Fig. 4A and Fig. S9), indicating that the fluctuations observed under 2.5 M NaCl were not reflected in the M2G accumulation. Thus, it is possible that the rhythm peaking at LL16-20 under 2.5 M NaCl was not a circadian oscillation, but a transient induction due to salt stress. If so, the number of genes actually undergoing circadian oscillation under 2.5 M NaCl conditions was very small. The results of Fig. 3A, in which the phase of circadian gene expression with 0.5 M NaCl was perturbed with 2.5 M NaCl, suggest that high salinity destabilizes the circadian clock in *Halothece*.

The question arose as to whether the central oscillator formed by the KaiABC protein was affected by high NaCl concentrations. It is known that the phosphorylation rhythm of KaiC is produced by the regulation of KaiA and KaiB^24,25^, and this was analyzed in *Halothece* by Western blotting (Fig. S10). Under both NaCl conditions, the KaiC phosphorylation level was always high; under 0.5 M NaCl conditions, the ratio of phosphorylation was found to be oscillating with peaks at LL10 and 34, while under 2.5 M NaCl conditions, the amplitude of the rhythm decreased. This result suggests that interactions among Kai proteins might be affected under high-salinity conditions. It would be interesting to investigate the relationship between the amplitude of the KaiC phosphorylation rhythm and the stability of the circadian rhythmicity in cyanobacteria.

## Conclusion

**Figure 5:**
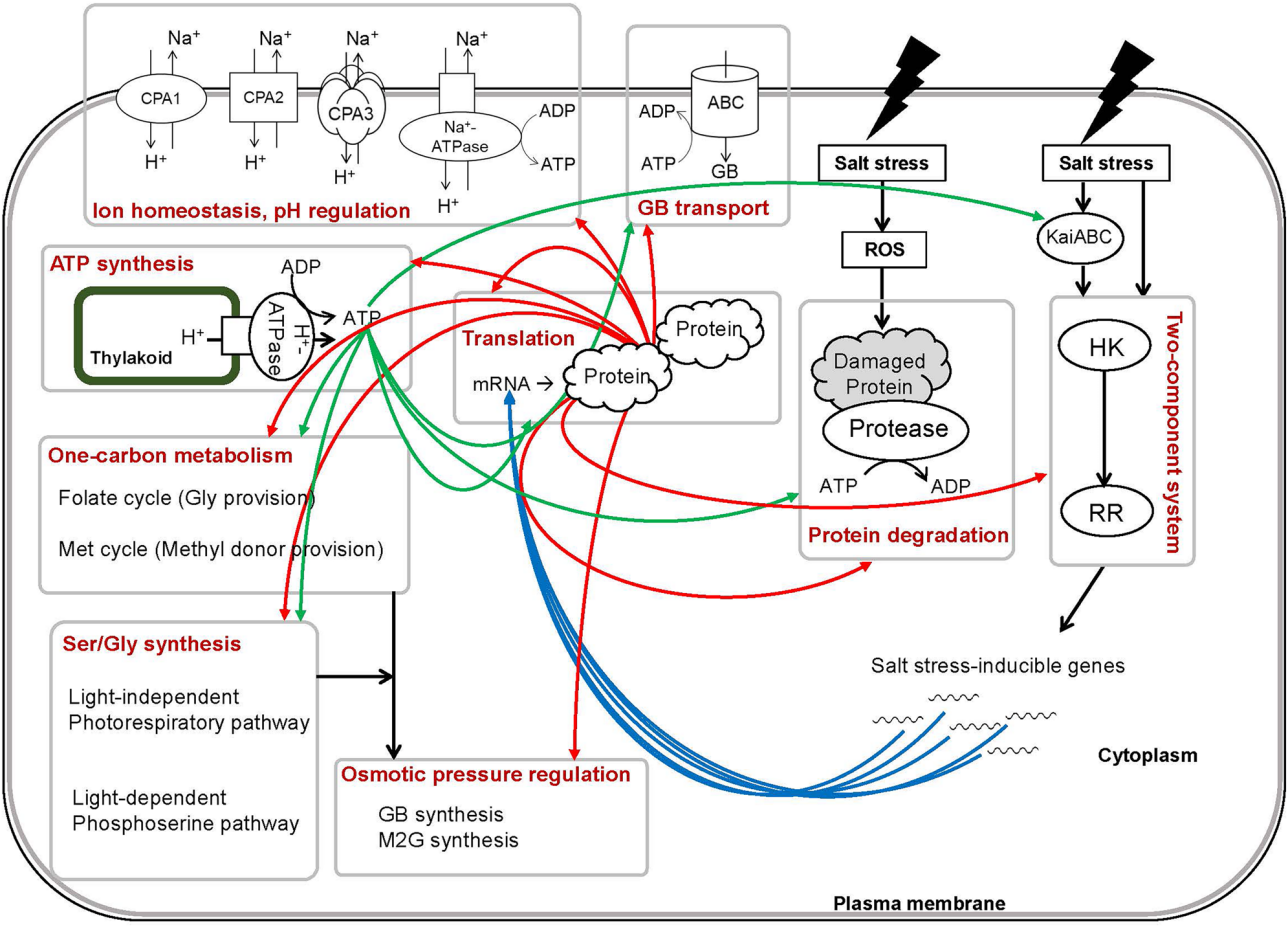
Transcriptional upregulation of signaling and biosynthetic pathways in high-salt-acclimated *Halothece* cells and their correlations. Blue, red, and green arrows indicate the flow of mRNA, protein, and ATP, respectively. See text and supplementary data for details.

Based on the detailed results of our RNA-seq analysis, we propose a molecular network of high-salinity acclimation mechanisms in halotolerant cyanobacterium. Molecular networks and regulatory mechanisms that are crucial for salt tolerance were found to be coordinated with the interlinking of signaling and metabolic pathways, such as ion homeostasis, energy production, and biosynthesis of secondary metabolites. The coordination of intracellular regulatory mechanisms with specific metabolic pathways is thought to maintain high levels of chemical reaction activity by activating the turnover of related proteins (Fig. 5). Furthermore, we found that external environmental stress, i.e., high-salinity conditions, significantly affected the circadian clock.

## Materials and methods

### Culture conditions for cyanobacteria

*Halothece* sp. PCC7418 was grown photoautotrophically in BG11 medium plus Turk Island salt solution, containing 0.5 M or 2.5 M NaCl^26^, under continuous light (LL) conditions of light-emitting diode of light intensity equivalent to 50 μmol m^−2^ s^−1^ at 30 °C for 10 days. To synchronize the circadian clock, the cultures acclimated to each NaCl condition were treated with two cycles of 12-h light 12-h dark (LD) alternation and then placed in LL conditions. The cells were cultivated and harvested after 4, 10, 16, 22, 28, and 34 h in LL conditions. The optical densities of the cultures were around 0.25 at 730 nm (OD_730_) before synchronization, and the values did not exceed 0.50 by the time sampling was completed. Biological duplicated samples were prepared and stored at − 80 °C.

### Extraction of total RNA

Total RNA was extracted utilizing TRIzol^Ⓡ^ LS Reagent (Thermo Fisher Scientific, Inc., Waltham, MA, USA), according to the manufacturer’s protocol. DNase I treatment was carried out to eliminate DNA contamination, using RNase-free recombinant DNase I (Takara Bio, Shiga, Japan). After treatment, RNA was recovered from the DNase I reaction mixture using phenol:chloroform extraction followed by ethanol precipitation. The RNA pellet was reconstituted with 50 µl of nuclease-free water (Takara Bio). RNA concentration was determined spectrophotometrically (BioSpec-nano, Kyoto, Japan) and visually confirmed using agarose gel electrophoresis.

### RNA sequencing (RNA-seq) analysis

RNA-seq was conducted by Bioengineering Lab. Co., Ltd., Kanagawa, Japan. The concentration of total RNA was measured using a Quantus Fluorometer and the QuantiFluor RNA System (Promega, Madison, WI, USA). The quality was then confirmed using the 5200 Fragment Analyzer System and the Agilent HS RNA Kit (Agilent Technologies, Inc., Santa Clara, CA, USA). For the preparation of libraries, MGIEasy RNA Directional Library Prep Set (MGI Tech Co., Ltd., Shenzhen, China) was utilized, according to the manufacturer’s protocol, following the removal of ribosomal RNA by using riboPool (siTOOLs Biotech, Munich, Germany). The concentration of the libraries was measured by utilizing a Qubit 3.0 Fluorometer and the dsDNA HS Assay Kit (Thermo Fisher Scientific, Inc.). Then, the quality of the libraries was confirmed using a Fragment Analyzer, the dsDNA 915 Reagent Kit/Agilent 2100 Bioanalyzer, and the High Sensitivity DNA Kit (Agilent Technologies). Following circularization using the MGIEasy Circularization Kit (MGI Tech Co., Ltd.), DNA nanoballs were prepared by utilizing the DNBSEQ-G400 RS High Throughput Sequencing Kit (MGI Tech Co., Ltd.). RNA sequencing was performed on a DNBSEQ-G400 (MGI Tech Co., Ltd.), using paired-end 200-bp sequencing.

For data analysis, the relative transcript expression level was normalized as TPM. The TPM values (n = 2) of LL 16, 22, 28, and 34 under low (0.5 M) or high (2.5 M) NaCl conditions were averaged, and DEGs between these conditions were extracted. Fold-change threshold values of 2 and 0.5 were utilized for the extraction of upregulated and downregulated genes, respectively.

### Detecting circadian cycling genes

We extracted statistically-significant cycling genes based on the normalized TPM time courses. The two indices for circadian cycling genes, “amplitude” and “correlation *p* value” were calculated from a time course of TPM data for each gene. “Amplitude” was defined as the standard deviation of time-course data and indicates how evident the gene fluctuation is. “Correlation *p* value” indicates how close the expression profile is to sinusoid. The value of Pearson’s correlation between the expression profile and the best-fit sinusoidal curve with a period of 24 h was computed. The best-fit sinusoidal curve was selected from 60 sinusoidal functions with different phases, which produced the maximum correlation value. Next, we generated 106 random time-course data series and then obtained the distribution that the maximum correlation values of the random time-course data obeyed. Based on this distribution, we calculated the “correlation *p* value”, the probability that a maximum correlation value from virtual random time-course exceeds the one from actual time-course. Because we had two independent TMP time courses, we considered the gene was undergoing circadian cycling when the amplitude exceeded 0.1 and the correlation *p* value was < 0.1 for both experiments. These filtering procedures and the threshold values used were identical to those used in a previous study^27^.

### GO analysis

*Halothece* gene sequences were compared with the Gene Ontology database (http://geneontology.org/) using BlastX, and a GO term was assigned to each sequence (E-value < *e*^5^). Then, Fisher’s exact probability test was performed on the DEGs.

### Other methods

Semi-quantitative reverse transcriptase PCR (RT-PCR) was performed as previously described^28^, using 1 μg of total RNA. The RT-PCR primers and PCR cycle number for each gene are shown in Table S2. M2G measurement was performed as previously described^4^.

## Supplementary Information

Supplementary Information 1.

Supplementary Information 2.

## Data Availability

All data generated or analyzed during this study are included in this published article (and its Supplementary Information files). The sequence data of RNA-seq analysis has been deposited in the Gene Expression Omnibus (GEO), https://www.ncbi.nlm.nih.gov/geo/ (accession no. GSE205640).
