# Supplementary material for: Global transcriptional and circadian regulation in a halotolerant cyanobacterium Halothece sp. PCC7418
Source: Sci Rep. 2022 Aug 12;12:13190. doi: 10.1038/s41598-022-17406-6 (PMC9374696; doi:10.1038/s41598-022-17406-6)
Supplement: Supplementary file 2 — Supplementary Information 2. [file 41598_2022_17406_MOESM2_ESM.docx]

**Supplemental Information**

Global transcriptional and circadian regulation in a halotolerant cyanobacterium *Halothece* sp. PCC7418

Rungaroon Waditee-Sirisattha^1,^*, Hiroshi Ito^2,^*, and Hakuto Kageyama^3,4,^*

1. Department of Microbiology, Faculty of Science, Chulalongkorn University, Phayathai Road, Pathumwan, Bangkok 10330, Thailand.

2. Faculty of Design, Kyushu University, Fukuoka, 815-8540, Japan.

3. Department of Chemistry, Faculty of Science and Technology, Meijo University, 1-501 Shiogamaguchi, Tenpaku-ku, Nagoya, Aichi 468-8502, Japan.

4. Graduate School of Environmental and Human Sciences, Meijo University, 1-501 Shiogamaguchi, Tenpaku-ku, Nagoya, Aichi 468-8502, Japan.

*To whom correspondence should be Rungaroon Waditee-Sirisattha, Hiroshi Ito, and Hakuto Kageyama.

Email: Rungaroon.W@chula.ac.th, hito@design.kyushu-u.ac.jp, or kageyama@meijo-u.ac.jp.

**
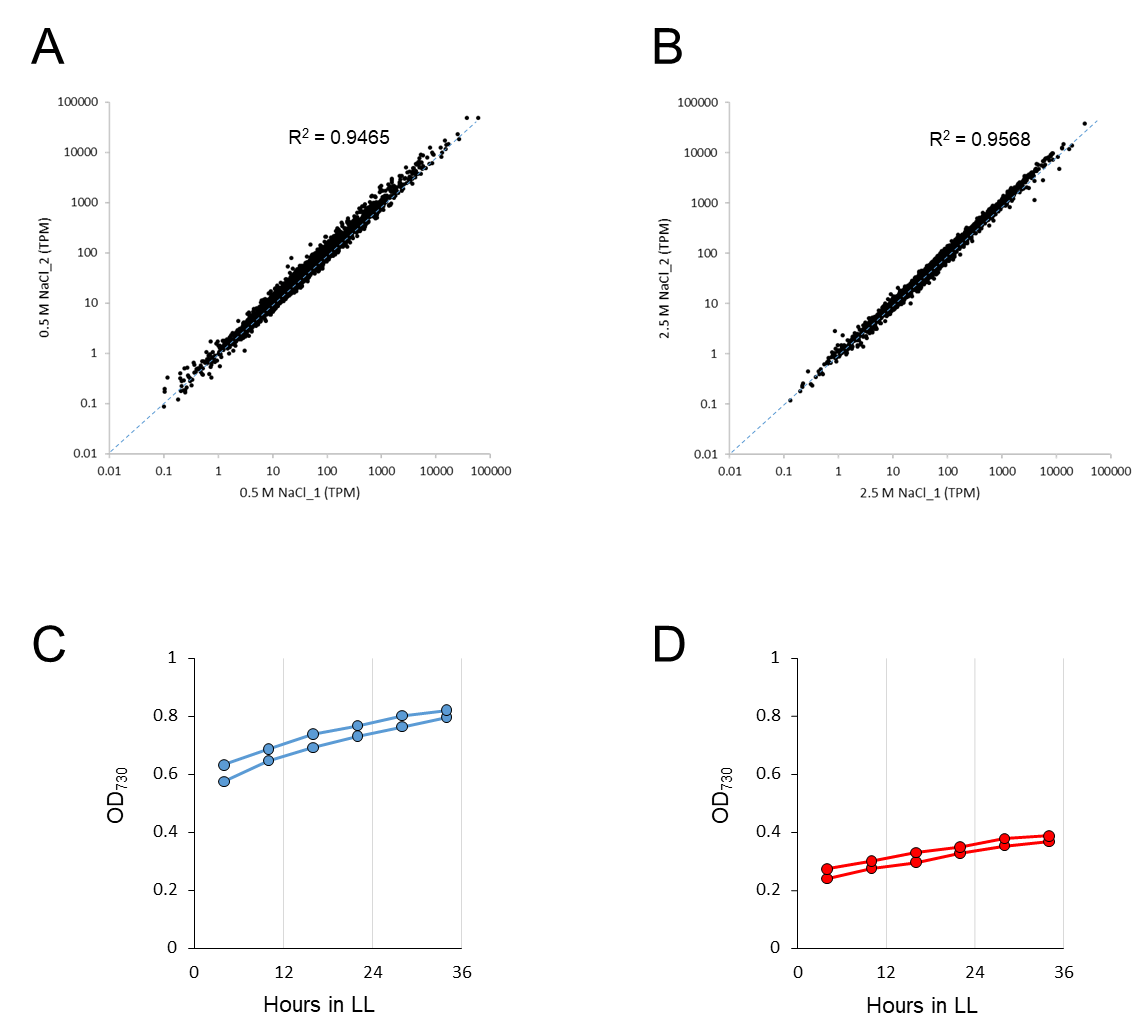
**

**Figure S1.** Scatter plots of transcriptional expression data for replicates of low- (A) and high- (B) salt-acclimated *Halothece* showing a high degree of correlation. (C) and (D) Growth of *Halothece* cultures investigated in this study under 0.5 M (C) and 2.5 M (D) NaCl conditions. The absorbance of cultures was measured at 730 nm at each sampling time.


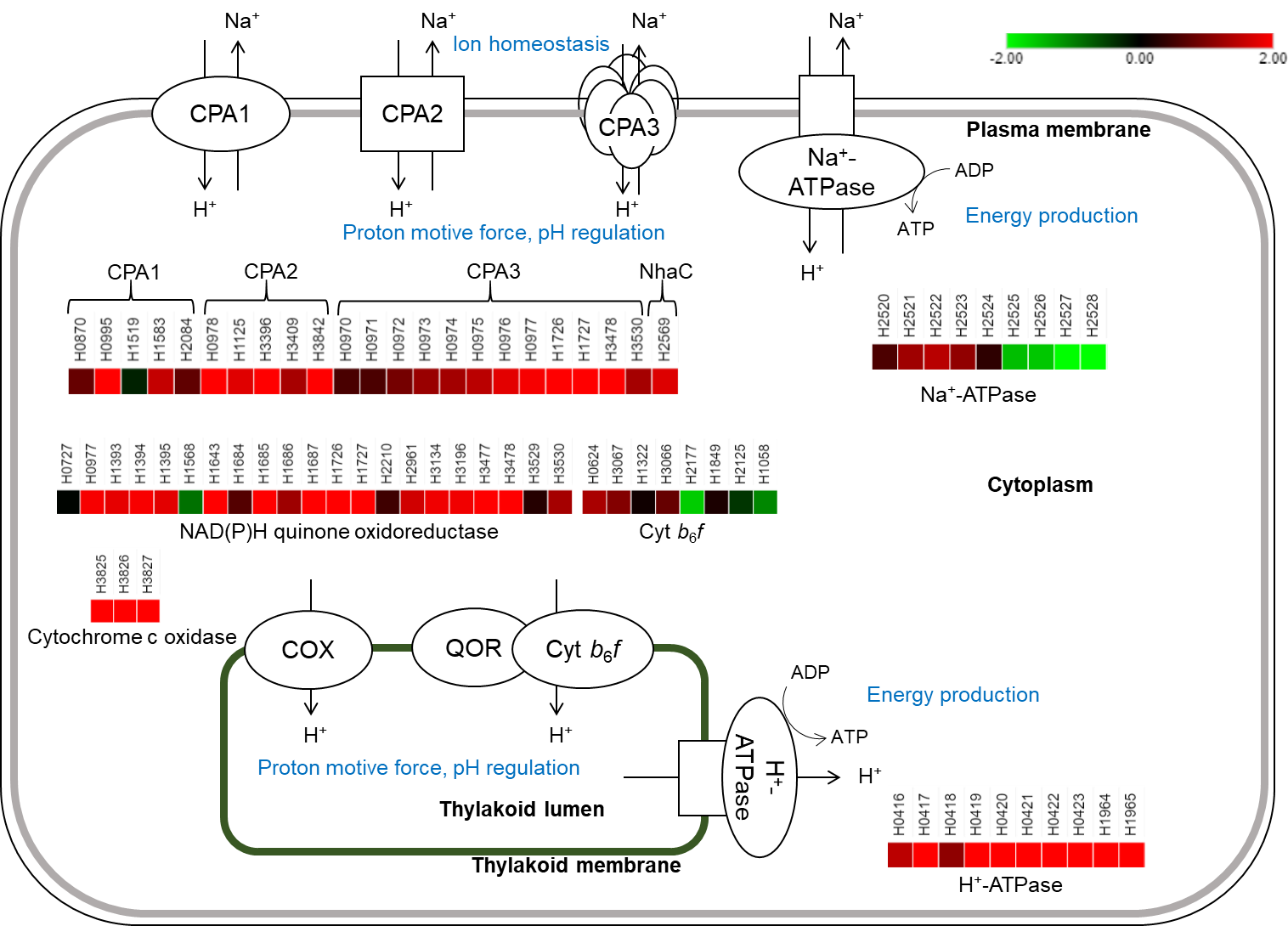


**Figure S2.** Schematic diagram of the factors related to ion homeostasis and energy production. The heatmaps illustrate the expression of related genes. The images of the heatmaps were created by using Morpheus (https://software.broadinstitute.org/morpheus). Abbreviations: CPA, cation/proton antiporter; QOR, NAD(P)H quinone oxidoreductase; Cyt*b*_6_*f*, cytochrome *b*_6_*f* complex; COX, cytochrome c oxidase.


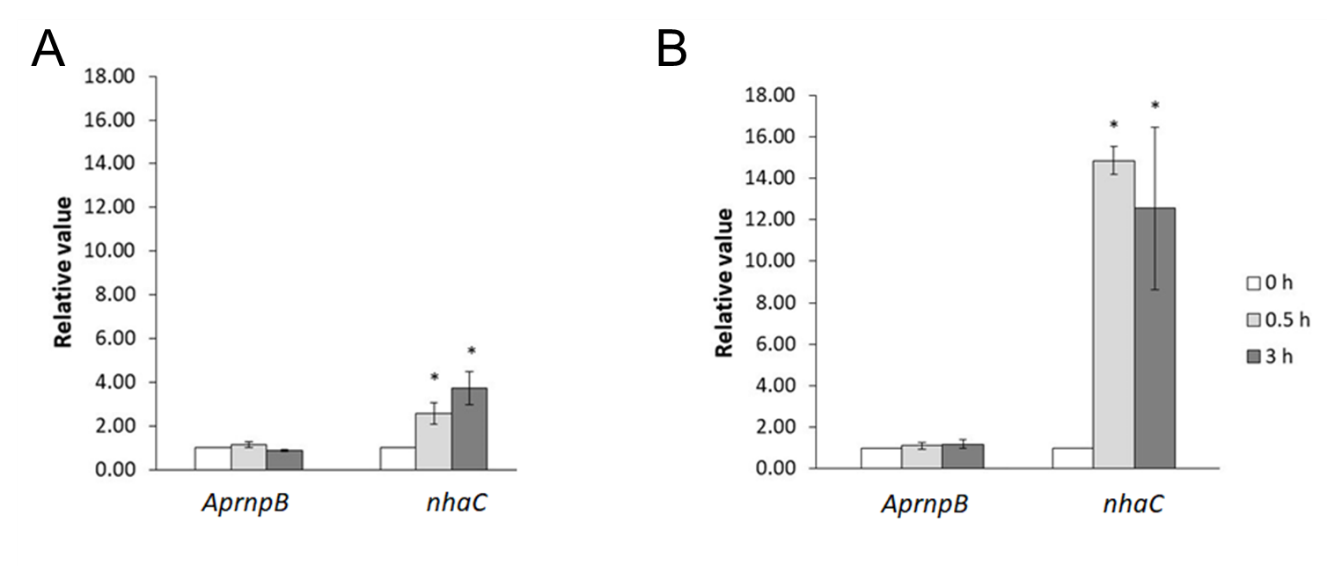


**Figure S3.** Expression of *nhaC* in *Halothece* 7418 cells. Cells were collected at 0, 0.5, and 3 h of exposure to salinity stress (A) or alkaline stress (B). Semiquantitative RT-PCR analysis was performed. PCR products were subjected to electrophoresis, followed by calculation of relative values of the amount of DNA fragments. Ribonuclease gene (*H0895*) was used as an internal control. Data are the means ± standard deviations of three independent experiments. Asterisks indicate significant difference (*P* < 0.05) from the values at time zero. The detailed methods are shown below.

**Stress treatments**

*Halothece* cells were photoautotrophically cultured at 30ºC in BG11 media plus Turk Island salt solution containing 0.5 M NaCl under continuous cool white fluorescent light intensity of 70 µE m^-2^ s^-1^. For salt-stress treatment, the cells were cultured to the exponential growth phase (OD730≅0.8) in BG11 media plus Turk Island salt solution containing 0.5 M NaCl. Cells were then harvested from 100 ml cultures by centrifugation and immediately transferred into the same volume of fresh medium containing 2.0 M NaCl. The cells treated under salt up-shock condition were harvested at 0, 0.5 and 3 hours.

For alkaline stress, the same volume scale as described in salt-stress treatment was performed. The cells were firstly cultured to the exponential growth phase (OD730≅0.8) in BG11 media plus Turk Island salt solution containing 0.5 M NaCl. Cells were then harvested by centrifugation and immediately transferred into the BG11 media plus Turk Island salt solution containing 0.5 M NaCl in which pH was adjusted to 10 by using 6 M NaOH. The cells treated under alkaline-stress condition were harvested at 0, 0.5 and 3 hours.

**Reverse transcription-PCR (RT-PCR) analysis**

Total RNA was extracted with TRIzol® reagent (Invitrogen, USA) according to the manufacturer’s instructions. RNA concentration and quality were determined using a Nanodrop 2000 (Thermo Scientific, USA) and gel electrophoresis, respectively. Five µg of total RNA was utilized as a template for cDNA synthesis with the SuperScript® III first-strand synthesis system (Invitrogen, USA). A specific primer pair for the *nhaC* gene (*H2569*); forward 5’ GTTAGGGAACATGATGGAG 3’ and reverse 5’ GGCATTGATAAACAAGCAG 3’ was used. A primer pair for the ribonuclease gene (*H0895*) gene was used as an internal control ^1^. The mRNA expression level was quantitated using Image LabTM 3.0 software (http://www.bio-rad.com/en-ch/product/image-lab-software). For statistical significance, the difference between control and stress-treated condition was assessed using one sample Student’s *t*-test. *P* values<0.05 were considered significant.


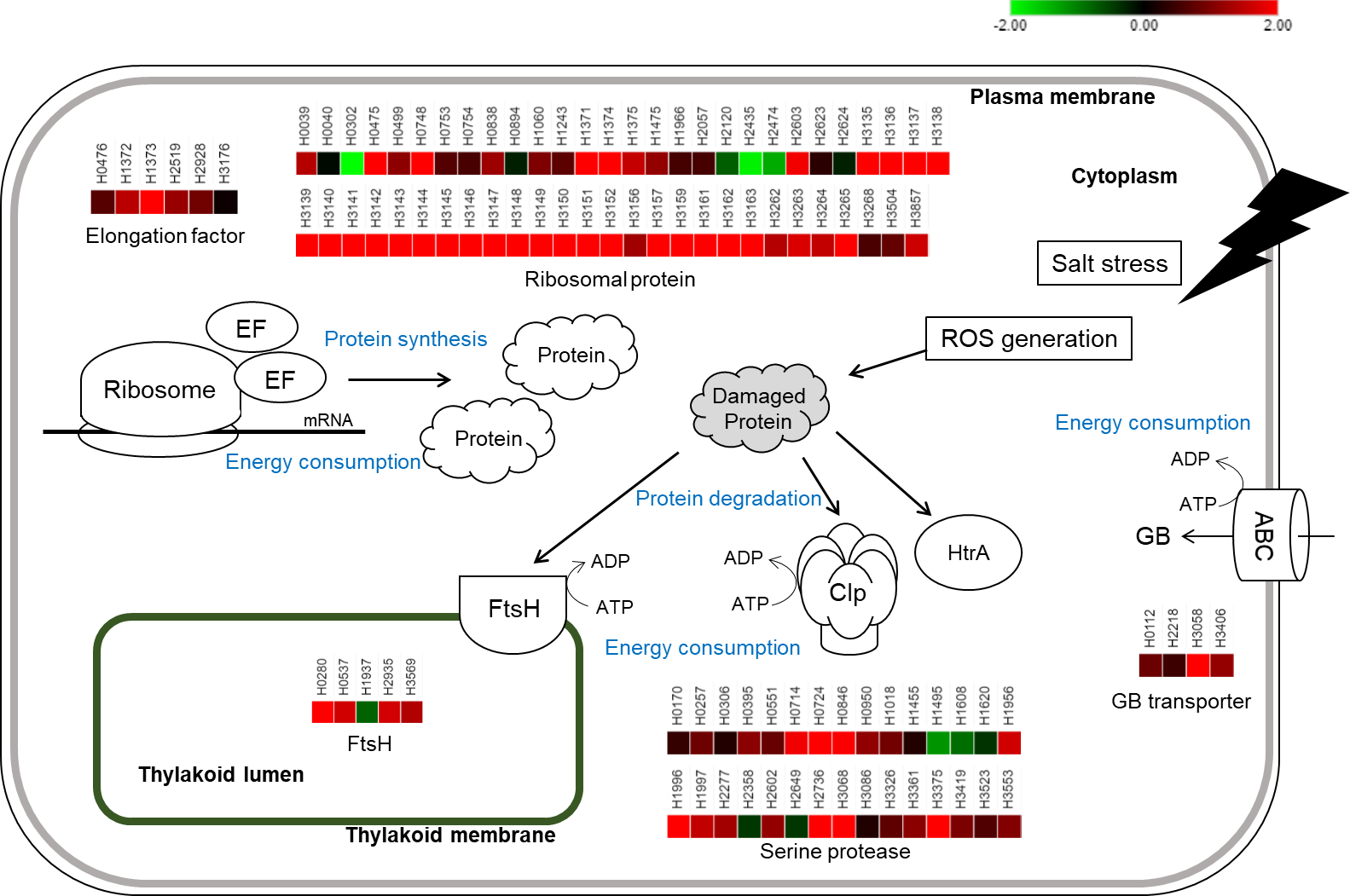


**Figure S4.** Schematic diagram of the factors related to protein homeostasis and GB transportation. The heatmaps illustrate the expression of related genes. The images of the heatmaps were created by using Morpheus (https://software.broadinstitute.org/morpheus). Abbreviations: ABC, ATP-binding-cassette transport system; EF, elongation factor.


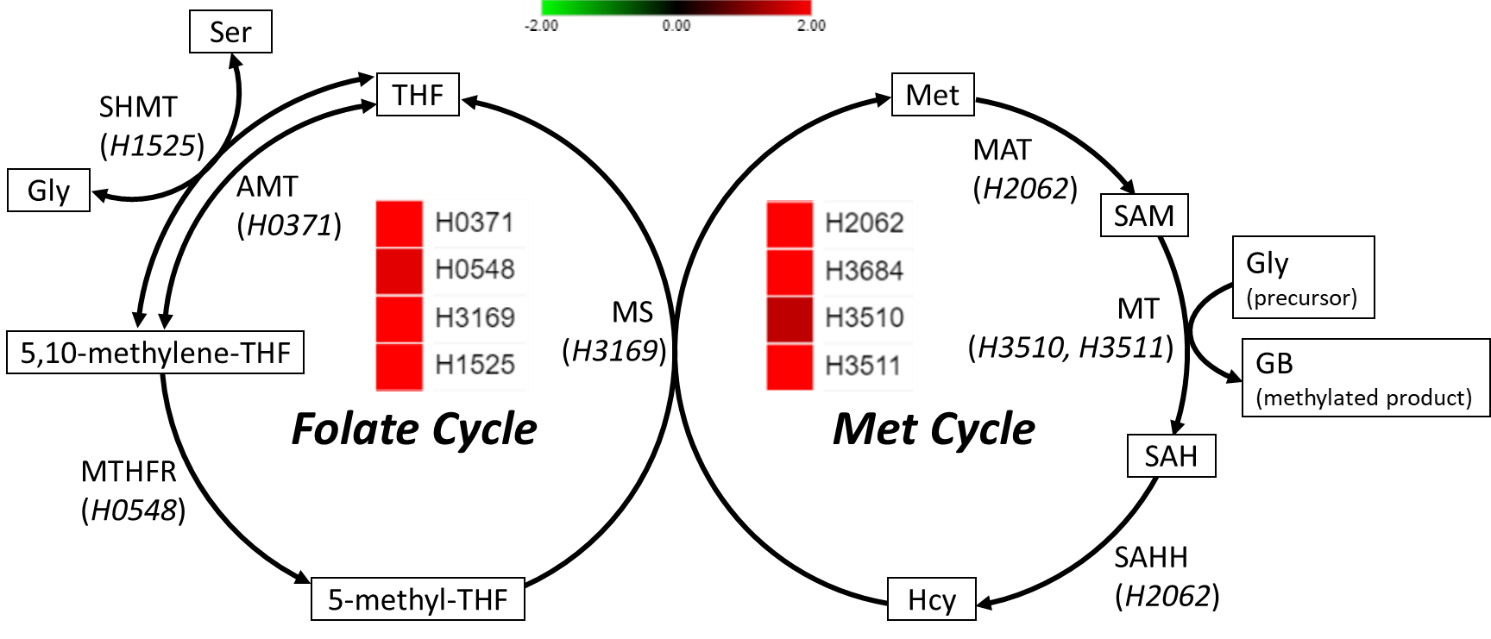


**Figure S5.** One-carbon metabolic pathway. The heatmaps illustrate the expression of related genes. The images of the heatmaps were created by using Morpheus (https://software.broadinstitute.org/morpheus). Abbreviations: THF, tetrahydrofolate; SAM, S-adenosylmethionine; SAH, S-adenosylhomocysteine; Hcy, homocysteine; GB, glycine betaine; SHMT, serine hydroxymethyltransferase; AMT, aminomethyltransferase; MTHFR, 5,10-methylene tetrahydrofolate reductase; MS, methionine synthase; MAT, methionine adenosyltransferase; MT, methyltransferase; SAHH, SAH hydrolase.


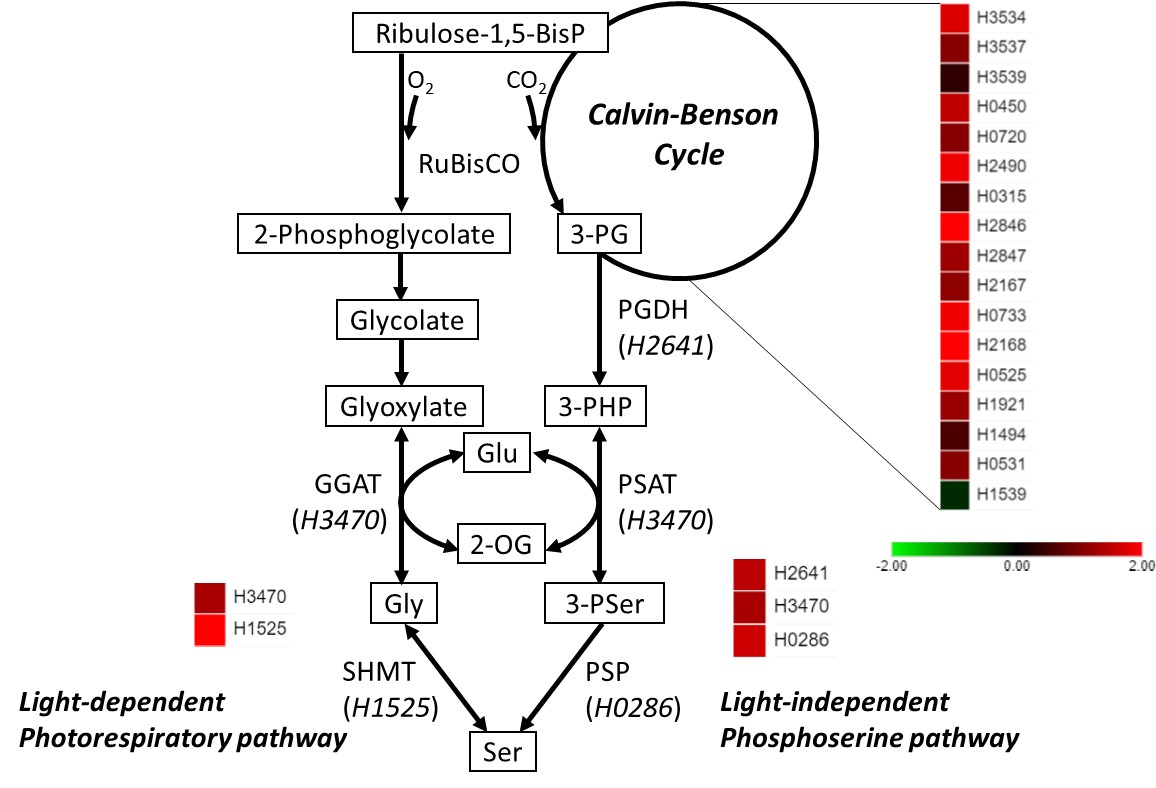


**Figure S6.** Serine biosynthetic pathway. The heatmaps illustrate the expression of related genes. The images of the heatmaps were created by using Morpheus (https://software.broadinstitute.org/morpheus). Abbreviations: 3-PG, 3-phosphoglycerate; 3-PHP, 3-phosphohydroxypyruvate; 3-PSer, 3-phosphoserine; 2-OG, 2-oxoglutarate; RuBisCO, ribulose 1, 5-bisphosphate carboxylase/oxygenase; PGDH, 3-phosphoglycerate dehydrogenase; PSAT, phosphoserine aminotransferase; PSP, phosphoserine phosphatase; SHMT, serine hydroxymethyltransferase; GGAT, glutamate:glyoxylate aminotransferase.


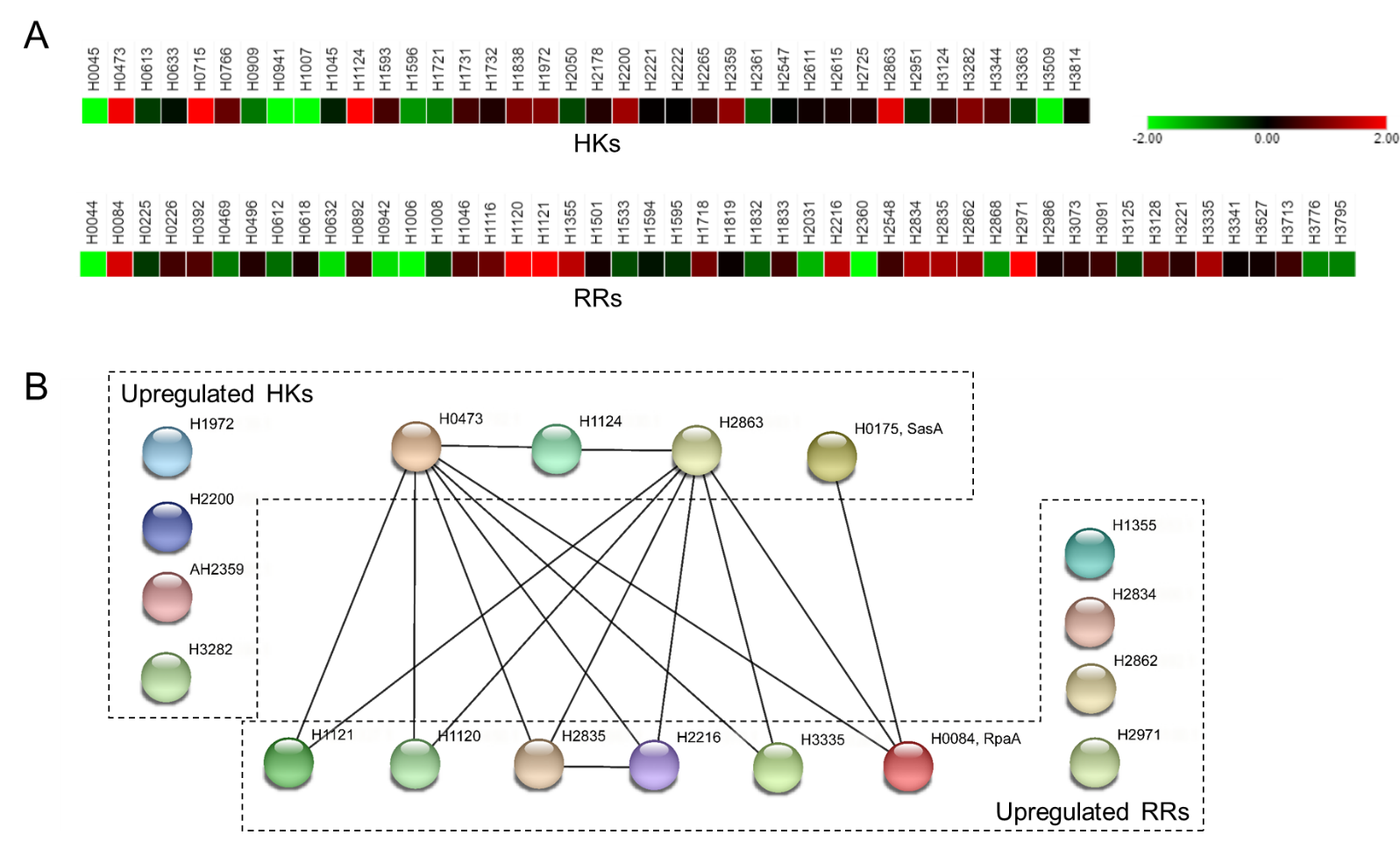


**Figure S7.** Expression and interaction of HKs and RRs. (A) The heatmaps illustrate the expression of HKs and RRs. The images of the heatmaps were created by using Morpheus (https://software.broadinstitute.org/morpheus). (B) Putative interaction network among HKs and RRs. The network was predicted by STRINGS (https://string-db.org/) using upregulated (log2FC ≥ 1) HKs and RRs. The settings used for the prediction were as follows. Network type: full STRING network, meaning of network edges: evidence, active interaction sources: experiments, minimum required interaction score: high confidence.


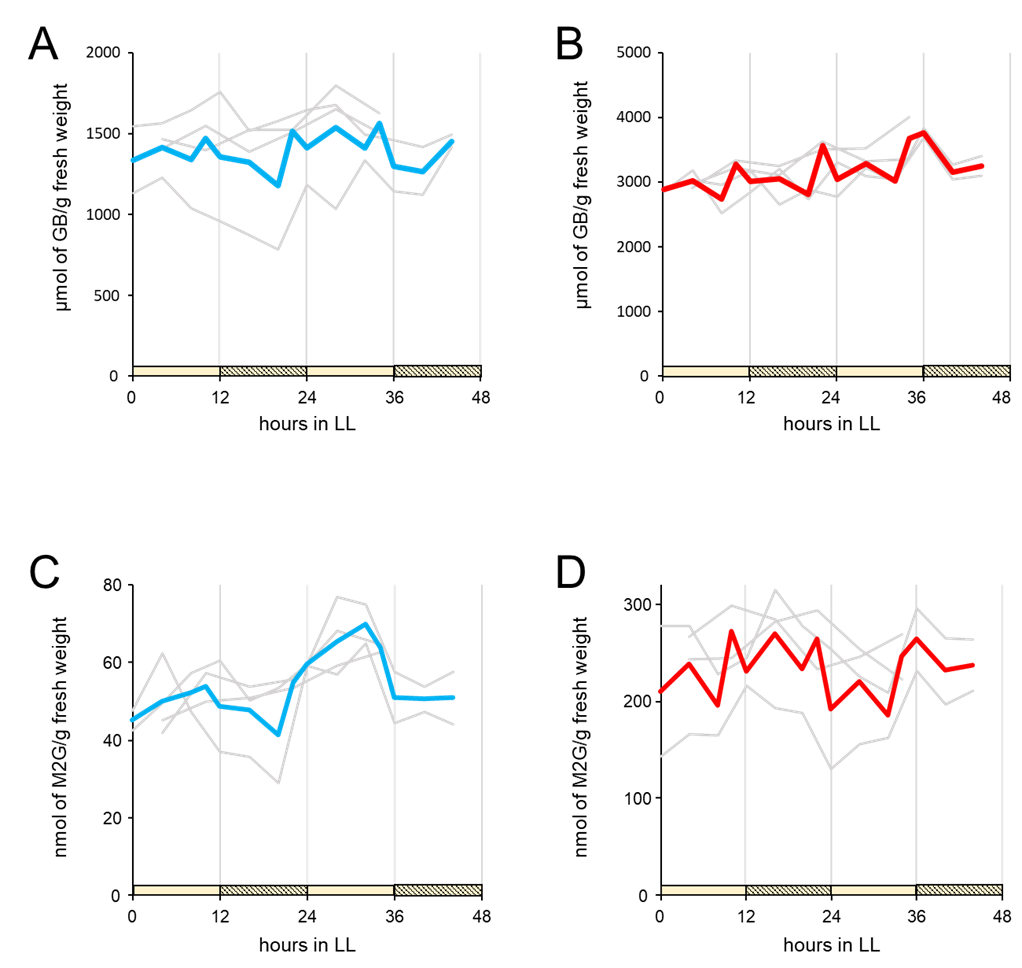


**Figure S8.** Temporal accumulation patterns of GB and M2G in *Halothece*. GB (A and B) and M2G (C and D) were extracted from *Halothece* and quantified according to a previously described protocol ^2^. The blue (A and C) and red (B and D) lines indicate accumulation profiles under conditions of 0.5 and 2.5 M NaCl, respectively, from four independent experiments (gray lines).


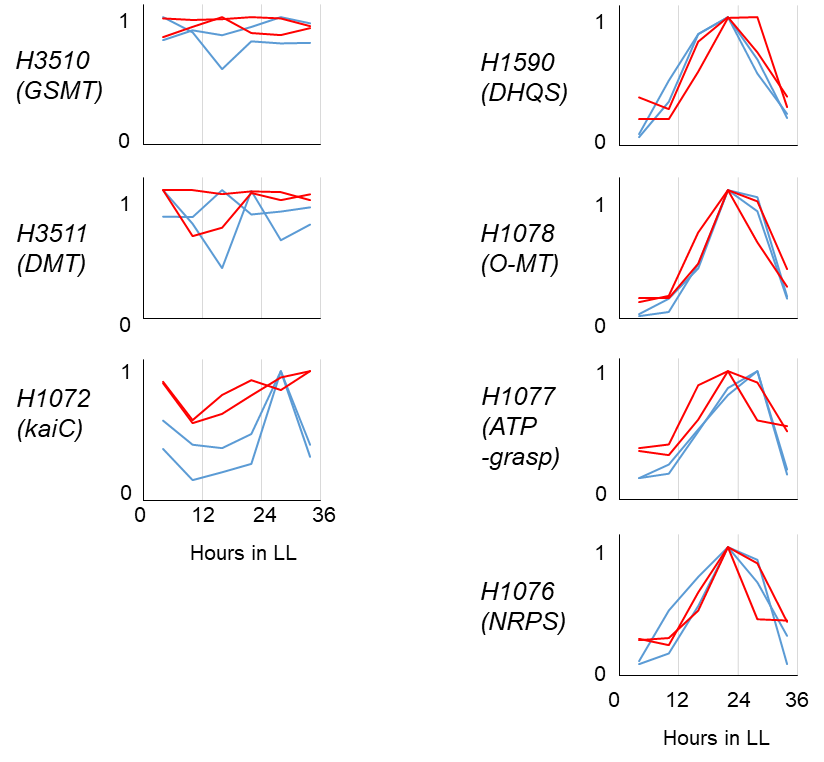


**Figure S9.** Semi-quantitative RT-PCR analysis for representative *Halothece* genes. The blue and red lines indicate expression profiles under conditions of 0.5 and 2.5 M NaCl, respectively, from two independent experiments. Relative values are shown, and the time when the intensity peaked was adjusted to 1.


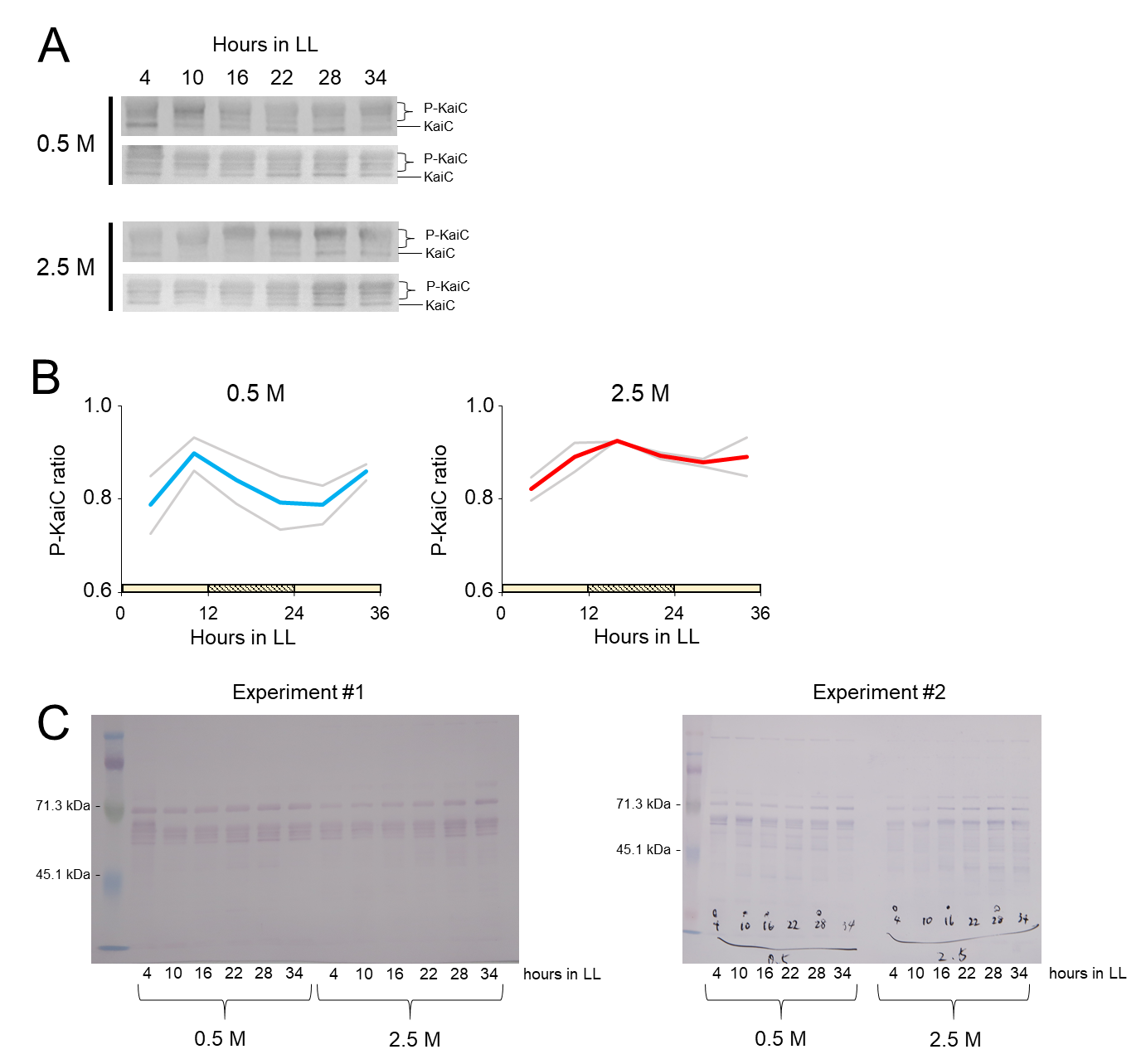


**Figure S10.** Temporal phosphorylation patterns of KaiC in *Halothece*. Total soluble protein was extracted from sampled *Halothece* cells as previously described ^3^, using buffer containing 100 mM Tris-HCl (pH 8.0), 2 mM EDTA, and protease inhibitors (Protease Inhibitor Cocktail Set III DMSO Solution (EDTA-free), Fujifilm Wako Chemicals, Osaka Japan). The protein concentration was determined using the Bradford method. For SDS-PAGE (8.5% gel), 2.5 µl total protein was loaded onto each lane. After blotting, KaiC was detected with anti-KaiC antiserum, using a 1:500-fold dilution. The antiserum was kindly gifted by Prof. Takao Kondo (Nagoya University). This antiserum was prepared by immunization of a rabbit with recombinant KaiC of *Synechococcus elongatus* PCC7942. Note that identity between *Halothece* KaiC and *Synechococcus* KaiC was 78% (Fig. S11). Antibody-bound proteins were visualized by a colorimetric method using alkaline phosphatase-conjugated secondary antibody (A). Original blots are presented in the panel (C). (B) Relative values of KaiC phosphorylation patterns. The blue and red lines indicate the average values of two independent experiments (gray lines). (C) Original blots of the panel (A).

**Figure S11.** Alignment of KaiC of *Halothece* and *Synechococcus elongatus* PCC7942.

Table S1. Number of up- and down-regulated genes in high-salt-acclimated cyanobacteria. The absolute values of log2|FC| ≥ 2 or ≥ 1 were used as the thresholds to identify significant DEGs.

| Strain | NaCl conc. vs control | Up | Down | Total | Threshold | Method | Ref. |
| --- | --- | --- | --- | --- | --- | --- | --- |
| *Halothece* sp. PCC7418 | 2.5 M vs 0.5 M | 1368 | 446 | 3843 | log2\|FC\| ≥ 1 | RNA-seq | This study |
| *Halothece* sp. PCC7418 | 2.5 M vs 0.5 M | 402 | 93 | 3843 | log2\|FC\| ≥ 2 | RNA-seq | This study |
| *Synechocystis* sp. PCC6803 | 0.5 M vs 0 M | 147 | 228 | 3079 | log2\|FC\| ≥ 1 | DNA microarray | ^4^ |
| *Prochlorococcus* AS9601 | 5% w/v vs 3.8% w/v | 31 | 38 | 1959 | log2\|FC\| ≥ 2 | RNA-seq | ^5^ |

Table S2. List of DEGs in high-salt-acclimated *Halothece* that were discussed in the Results and Discussion section.

| Gene ID | Definition | Log2FC |
| --- | --- | --- |
| **CPAs** | | |
| *H0870* | CPA1 | 0.80 |
| *H0995* | CPA1 | 2.66 |
| *H1519* | CPA1 | -0.26 |
| *H1583* | CPA1 | 1.54 |
| *H2084* | CPA1 | 0.75 |
| *H0978* | CPA2 | 2.55 |
| *H1125* | CPA2 | 1.75 |
| *H3396* | CPA2 | 2.07 |
| *H3409* | CPA2 | 1.31 |
| *H3842* | CPA2 | 3.07 |
| *H0970* | CPA3 | 0.58 |
| *H0971* | CPA3 | 0.58 |
| *H0972* | CPA3 | 0.92 |
| *H0973* | CPA3 | 1.20 |
| *H0974* | CPA3 | 1.28 |
| *H0975* | CPA3 | 1.46 |
| *H0976* | CPA3 | 1.78 |
| *H0977* | CPA3 | 1.94 |
| *H1726* | CPA3 | 2.66 |
| *H1727* | CPA3 | 2.77 |
| *H2569* | NhaC | 1.73 |
|  | | |
| **Na^+^-ATPase** | | |
| *H2520* | F_0_F_1_ ATP synthase subunit gamma | 0.60 |
| *H2521* | alternate F_0_F_1_ ATPase, F_1_ subunit alpha | 1.24 |
| *H2522* | F_0_F_1_ ATP synthase subunit delta | 1.44 |
| *H2523* | F_0_F_1_ ATP synthase subunit C | 1.14 |
| *H2524* | F_0_F_1_ ATP synthase subunit A | 0.36 |
| *H2525* | ATP synthase subunit I | -1.49 |
| *H2526* | AtpZ/AtpI family protein | -1.56 |
| *H2527* | F_0_F_1_ ATP synthase subunit epsilon | -2.21 |
| *H2528* | F_0_F_1_ ATP synthase subunit beta | -2.31 |
|  | | |
| **H^+^-ATPase** | | |
| *H0416* | ATP synthase subunit I | 1.46 |
| *H0417* | F_0_F_1_ ATP synthase subunit A | 2.21 |
| *H0418* | ATP synthase F_0_ subunit C | 1.12 |
| *H0419* | F_0_F_1_ ATP synthase subunit B' | 3.54 |
| *H0420* | F_0_F_1_ ATP synthase subunit B | 3.50 |
| *H0421* | F_0_F_1_ ATP synthase subunit delta | 3.49 |
| *H0422* | F_0_F_1_ ATP synthase subunit alpha | 2.66 |
| *H0423* | F_0_F_1_ ATP synthase subunit gamma | 2.38 |
| *H1964* | F_0_F_1_ ATP synthase subunit beta | 2.24 |
| *H1965* | F_0_F_1_ ATP synthase subunit epsilon | 2.11 |
|  | | |
| **Cytochrome c oxidase** | | |
| H3825 | heme-copper oxidase subunit III | 2.27 |
| H3826 | cytochrome c oxidase subunit I | 2.79 |
| H3827 | cytochrome c oxidase subunit II | 2.40 |
|  | | |
| **NAD(P)H-quinone oxidoreductase** | | |
| *H0727* | NAD(P)H-quinone oxidoreductase subunit O | -0.03 |
| *H0977* | NADH-quinone oxidoreductase subunit K | 1.94 |
| *H1393* | NAD(P)H-quinone oxidoreductase subunit J | 1.80 |
| *H1394* | NADH-quinone oxidoreductase subunit NuoB | 1.94 |
| *H1395* | photosynthetic/respiratory NAD(P)H-quinone oxidoreductase subunit C | 1.78 |
| *H1568* | NAD(P)H-quinone oxidoreductase subunit L | -0.89 |
| *H1643* | NAD(P)H-quinone oxidoreductase subunit 4 | 3.88 |
| *H1684* | NADH-quinone oxidoreductase subunit NuoK | 0.66 |
| *H1685* | NADH-quinone oxidoreductase subunit J | 2.22 |
| *H1686* | NAD(P)H-quinone oxidoreductase subunit I | 1.24 |
| *H1687* | NADH-quinone oxidoreductase subunit NuoH | 2.42 |
| *H1726* | NAD(P)H-quinone oxidoreductase subunit 5 | 2.66 |
| *H1727* | NAD(P)H-quinone oxidoreductase subunit 4 | 2.77 |
| *H2210* | NAD(P)H-quinone oxidoreductase subunit M | 0.50 |
| *H2961* | NAD(P)H-quinone oxidoreductase subunit H | 1.63 |
| *H3134* | NAD(P)H-quinone oxidoreductase subunit N | 1.88 |
| *H3196* | NAD(P)H-quinone oxidoreductase subunit N | 1.89 |
| *H3477* | NADH-quinone oxidoreductase subunit M | 3.12 |
| *H3478* | NAD(P)H-quinone oxidoreductase subunit F | 2.52 |
| *H3529* | NADH-quinone oxidoreductase subunit M | 0.29 |
| *H3530* | NAD(P)H-quinone oxidoreductase subunit F | 1.29 |
|  | | |
| **Cytochrome *b_6_f* complex** | | |
| *H0624* | apocytochrome f PetA | 1.33 |
| *H3067* | cytochrome *b_6_* PetB | 1.03 |
| *H1322* | cytochrome *b_6_f* complex iron-sulfur subunit PetC | 0.12 |
| *H3066* | cytochrome *b_6_f* complex subunit IV PetD | 0.80 |
| *H2177* | cytochrome *b_6_f* complex subunit PetG | -1.60 |
| *H1849* | cytochrome *b_6_f* complex subunit PetL | 0.21 |
| *H2125* | cytochrome *b_6_f* complex subunit 7 PetM | -0.42 |
| *H1058* | cytochrome *b_6_f* complex subunit PetN | -1.06 |
|  | | |
| **Ribosomal protein** | | |
| *H0039* | 50S ribosomal protein L21 | 1.42 |
| *H0040* | 50S ribosomal protein L27 | -0.07 |
| *H0302* | 50S ribosomal protein L28 | -2.02 |
| *H0475* | 30S ribosomal protein S2 | 2.24 |
| *H0499* | 30S ribosomal protein S16 | 1.11 |
| *H0748* | 30S ribosomal protein S15 | 2.17 |
| *H0753* | 30S ribosomal protein S18 | 0.67 |
| *H0754* | 50S ribosomal protein L33 | 0.57 |
| *H0838* | 30S ribosomal protein S4 | 1.23 |
| *H0894* | 50S ribosomal protein L34 | -0.23 |
| *H1060* | 30S ribosomal protein S1 | 0.99 |
| *H1243* | 50S ribosomal protein L9 | 0.72 |
| *H1371* | 30S ribosomal protein S10 | 2.13 |
| *H1374* | 30S ribosomal protein S7 | 2.97 |
| *H1375* | 30S ribosomal protein S12 | 1.54 |
| *H1475* | 30S ribosomal protein S6 | 1.15 |
| *H1966* | 30S ribosomal protein PSRP-3 | 0.54 |
| *H2057* | 30S ribosomal protein S1 | 0.55 |
| *H2120* | 30S ribosomal protein S20 | -0.73 |
| *H2435* | 30S ribosomal protein S21 | -1.89 |
| *H2474* | 50S ribosomal protein L32 | -1.34 |
| *H2603* | 50S ribosomal protein L25/general stress protein Ctc | 1.90 |
| *H2623* | 50S ribosomal protein L35 | 0.34 |
| *H2624* | 50S ribosomal protein L20 | -0.25 |
| *H3135* | 50S ribosomal protein L3 | 3.03 |
| *H3136* | 50S ribosomal protein L4 | 2.91 |
| *H3137* | 50S ribosomal protein L23 | 2.71 |
| *H3138* | 50S ribosomal protein L2 | 2.99 |
| *H3139* | 30S ribosomal protein S19 | 2.42 |
| *H3140* | 50S ribosomal protein L22 | 2.93 |
| *H3141* | 30S ribosomal protein S3 | 3.14 |
| *H3142* | 50S ribosomal protein L16 | 3.01 |
| *H3143* | 50S ribosomal protein L29 | 3.04 |
| *H3144* | 30S ribosomal protein S17 | 3.13 |
| *H3145* | 50S ribosomal protein L14 | 3.08 |
| *H3146* | 50S ribosomal protein L24 | 3.07 |
| *H3147* | 50S ribosomal protein L5 | 2.78 |
| *H3148* | 30S ribosomal protein S8 | 2.69 |
| *H3149* | 50S ribosomal protein L6 | 2.76 |
| *H3150* | 50S ribosomal protein L18 | 2.55 |
| *H3151* | 30S ribosomal protein S5 | 2.52 |
| *H3152* | 50S ribosomal protein L15 | 2.19 |
| *H3156* | 30S ribosomal protein S13 | 1.25 |
| *H3157* | 30S ribosomal protein S11 | 2.81 |
| *H3159* | 50S ribosomal protein L17 | 2.54 |
| *H3161* | 50S ribosomal protein L13 | 3.32 |
| *H3162* | 30S ribosomal protein S9 | 2.86 |
| *H3163* | 50S ribosomal protein L31 | 2.62 |
| *H3262* | 50S ribosomal protein L7/L12 | 1.41 |
| *H3263* | 50S ribosomal protein L10 | 1.71 |
| *H3264* | 50S ribosomal protein L1 | 1.53 |
| *H3265* | 50S ribosomal protein L11 | 2.05 |
| *H3268* | 50S ribosomal protein L19 | 0.59 |
| *H3504* | 30S ribosomal protein S14 | 0.77 |
| *H3857* | 30S ribosomal protein S21 | 1.60 |
|  | | |
| **Elongation factor** | | |
| *H0476* | translation elongation factor Ts | 0.68 |
| *H1372* | elongation factor Tu (*tuf*) | 1.45 |
| *H1373* | elongation factor G (*fusA*) | 2.52 |
| *H2519* | elongation factor G | 1.19 |
| *H2928* | elongation factor P | 0.88 |
|  | | |
| **FtsH** | | |
| *H0280* | ATP-dependent metallopeptidase FtsH/Yme1/Tma family protein | 2.38 |
| *H0537* | ATP-dependent metallopeptidase FtsH/Yme1/Tma family protein | 1.64 |
| *H1937* | ATP-dependent metallopeptidase FtsH/Yme1/Tma family protein | -0.77 |
| *H2935* | ATP-dependent metallopeptidase FtsH/Yme1/Tma family protein | 1.65 |
| *H3569* | ATP-dependent metallopeptidase FtsH/Yme1/Tma family protein | 1.39 |
|  | | |
| **Serine protease** | | |
| *H0170* | peptidase S8 | 0.41 |
| *H0257* | S9 family peptidase | 0.85 |
| *H0306* | signal peptidase I | 0.27 |
| *H0395* | trypsin-like peptidase domain-containing protein | 1.09 |
| *H0551* | signal peptidase I | 0.81 |
| *H0714* | LD-carboxypeptidase | 1.88 |
| *H0724* | S9 family peptidase | 4.05 |
| *H0846* | S1 family peptidase | 1.97 |
| *H0950* | ATP-dependent Clp endopeptidase proteolytic subunit ClpP | 1.13 |
| *H1018* | S8 family serine peptidase | 0.89 |
| *H1455* | signal peptide peptidase SppA | 0.27 |
| *H1495* | S8 family serine peptidase | -1.17 |
| *H1608* | D-alanyl-D-alanine carboxypeptidase/D-alanyl-D-alanine-endopeptidase | -0.86 |
| *H1620* | trypsin-like peptidase domain-containing protein | -0.40 |
| *H1956* | PDZ domain-containing protein | 1.64 |
| *H1996* | ATP-dependent Clp protease proteolytic subunit | 2.16 |
| *H1997* | ATP-dependent Clp protease proteolytic subunit | 1.52 |
| *H2277* | rhomboid family intramembrane serine protease | 1.27 |
| *H2358* | trypsin-like peptidase domain-containing protein | -0.42 |
| *H2602* | PDZ domain-containing protein | 1.15 |
| *H2649* | rhomboid family intramembrane serine protease | -0.43 |
| *H2736* | rhomboid family intramembrane serine protease | 2.37 |
| *H3068* | PDZ domain-containing protein | 2.16 |
| *H3086* | nickel-type superoxide dismutase maturation protease | 0.26 |
| *H3326* | signal peptide peptidase SppA | 0.74 |
| *H3361* | LON peptidase substrate-binding domain-containing protein | 1.08 |
| *H3375* | ATP-dependent Clp endopeptidase proteolytic subunit ClpP | 2.07 |
| *H3419* | D-alanyl-D-alanine carboxypeptidase | 0.97 |
| *H3523* | S9 family peptidase | 0.63 |
| *H3553* | trypsin-like peptidase domain-containing protein (HtrA) | 1.02 |
|  | | |
| **GB transporter** | | |
| *H0112* | glycine betaine/L-proline ABC transporter substrate-binding protein ProX | 0.84 |
| *H2218* | glycine betaine/L-proline ABC transporter ATP-binding protein | 0.47 |
| *H3058* | BCCT family transporter | 2.07 |
| *H3406* | glycine betaine/L-proline ABC transporter substrate-binding protein ProX | 1.16 |
|  | | |
| **One-carbon metabolism** | | |
| *H0371* | aminomethyltransferase | 2.36 |
| *H0548* | methylenetetrahydrofolate reductase | 1.78 |
| *H3169* | methionine synthase | 2.60 |
| *H1525* | serine hydroxymethyltransferase | 2.69 |
| *H2062* | methionine adenosyltransferase | 2.57 |
| *H3684* | adenosylhomocysteinase | 2.52 |
| *H3510* | class I SAM-dependent methyltransferase | 1.50 |
| *H3511* | methyltransferase domain-containing protein | 2.44 |
|  | | |
| **Light-dependent phosphoserine pathway** | | |
| *H2641* | phosphoglycerate dehydrogenase | 1.46 |
| *H3470* | alanine--glyoxylate aminotransferase family protein | 1.32 |
| *H0286* | histidine phosphatase family protein | 1.59 |
|  | | |
| **Light-independent photorespiratory pathway** | | |
| *H3470* | alanine--glyoxylate aminotransferase family protein | 1.32 |
| *H1525* | serine hydroxymethyltransferase | 2.69 |
|  | | |
| **Calvin-Benson cycle** | | |
| *H3534* | ribulose bisphosphate carboxylase small subunit | 1.72 |
| *H3537* | form I ribulose bisphosphate carboxylase large subunit | 1.06 |
| *H3539* | ribulose bisphosphate carboxylase small subunit | 0.38 |
| *H0450* | phosphoglycerate kinase | 1.49 |
| *H0720* | type I glyceraldehyde-3-phosphate dehydrogenase | 1.04 |
| *H2490* | type I glyceraldehyde-3-phosphate dehydrogenase | 1.87 |
| *H0315* | triose-phosphate isomerase | 0.69 |
| *H2846* | fructose-bisphosphate aldolase class I | 2.26 |
| *H2847* | fructose-bisphosphate aldolase class II | 1.23 |
| *H2167* | class 1 fructose-bisphosphatase | 1.11 |
| *H0733* | class II fructose-bisphosphatase | 1.90 |
| *H2168* | transaldolase | 2.20 |
| *H0525* | transketolase | 1.77 |
| *H1921* | ribose-5-phosphate isomerase RpiA | 1.20 |
| *H1494* | ribulose-phosphate 3-epimerase | 0.59 |
| *H0531* | phosphoribulokinase | 1.06 |
| *H1539* | phosphoribulokinase | -0.32 |
|  | | |
| **M2G synthetic pathway** | | |
| *H1590* | sedoheptulose 7-phosphate cyclase | 0.78 |
| *H1078* | class I SAM-dependent methyltransferase | 1.10 |
| *H1077* | ATP-grasp domain-containing protein | 0.43 |
| *H1076* | hypothetical protein | -0.96 |
|  | | |
| **Two-component system** | | |
| **Histidine kinase** | | |
| *H0045* | signal transduction histidine kinase | -2.59 |
| *H0473* | CheA signal transduction histidine kinase | 1.90 |
| *H0613* | multi-sensor hybrid histidine kinase | -0.46 |
| *H0633* | HAMP domain-containing histidine kinase | -0.12 |
| *H0715* | histidine kinase (SasA) | 2.49 |
| *H0766* | GAF sensor signal transduction histidine kinase | 0.80 |
| *H0909* | histidine kinase | -0.93 |
| *H0941* | sensor histidine kinase | -2.56 |
| *H1007* | hybrid sensor histidine kinase/response regulator | -2.60 |
| *H1045* | multi-sensor hybrid histidine kinase | -0.24 |
| *H1124* | hybrid sensor histidine kinase/response regulator | 3.10 |
| *H1593* | multi-sensor hybrid histidine kinase | 0.55 |
| *H1596* | Signal transduction histidine kinase | -1.16 |
| *H1721* | two-component sensor histidine kinase | -0.97 |
| *H1731* | multi-sensor hybrid histidine kinase | 0.52 |
| *H1732* | multi-sensor hybrid histidine kinase | 0.24 |
| *H1838* | CheA signal transduction histidine kinase | 0.99 |
| *H1972* | histidine kinase HAMP region domain protein | 1.02 |
| *H2050* | multi-sensor signal transduction histidine kinase (Hik33) | -0.55 |
| *H2178* | hybrid sensor histidine kinase/response regulator | 0.27 |
| *H2200* | PAS/PAC sensor signal transduction histidine kinase | 1.24 |
| *H2221* | PAS/PAC sensor hybrid histidine kinase | 0.03 |
| *H2222* | hybrid sensor histidine kinase/response regulator | 0.01 |
| *H2265* | GAF sensor signal transduction histidine kinase | 0.45 |
| *H2359* | multi-sensor signal transduction histidine kinase | 1.13 |
| *H2361* | multi-sensor signal transduction histidine kinase | -0.74 |
| *H2547* | Integral membrane sensor signal transduction histidine kinase | -0.02 |
| *H2611* | integral membrane sensor signal transduction histidine kinase | 0.12 |
| *H2615* | HAMP domain-containing histidine kinase | 0.18 |
| *H2725* | multi-sensor hybrid histidine kinase | 0.20 |
| *H2831* | CheA signal transduction histidine kinase | No data |
| *H2832* | CheA signal transduction histidine kinase | No data |
| *H2863* | signal transduction histidine kinase | 1.78 |
| *H2951* | multi-sensor hybrid histidine kinase (Hik16) | -0.37 |
| *H3124* | PAS/PAC sensor signal transduction histidine kinase | 0.41 |
| *H3282* | Putative CheA signal transduction histidine kinase | 1.03 |
| *H3344* | hybrid sensor histidine kinase/response regulator | 0.69 |
| *H3363* | GAF sensor signal transduction histidine kinase | -0.50 |
| *H3509* | HAMP domain-containing histidine kinase (Hik34) | -1.92 |
| *H3814* | multi-sensor hybrid histidine kinase | 0.11 |
|  | | |
| **Response regulators** | | |
| *H0044* | response regulator | -2.32 |
| *H0084* | response regulator transcription factor (RpaA) | 1.68 |
| *H0225* | response regulator receiver | -0.44 |
| *H0226* | response regulator | 0.52 |
| *H0392* | response regulator transcription factor | 0.63 |
| *H0469* | response regulator | -0.95 |
| *H0496* | response regulator receiver modulated diguanylate cyclase | 0.27 |
| *H0612* | response regulator transcription factor | -0.85 |
| *H0618* | response regulator | 0.21 |
| *H0623* | response regulator receiver domain | No data |
| *H0632* | response regulator transcription factor | -1.76 |
| *H0892* | response regulator | 0.47 |
| *H0942* | response regulator transcription factor | -1.75 |
| *H1006* | response regulator | -3.20 |
| *H1008* | response regulator receiver modulated CheB methylesterase | -0.55 |
| *H1046* | two component transcriptional regulator, winged helix family | 0.64 |
| *H1116* | response regulator transcription factor | 0.84 |
| *H1120* | response regulator | 3.64 |
| *H1121* | response regulator | 2.89 |
| *H1355* | response regulator transcription factor | 1.47 |
| *H1501* | response regulator | 0.16 |
| *H1533* | response regulator | -0.59 |
| *H1594* | EAL domain-containing response regulator | -0.15 |
| *H1595* | response regulator | -0.38 |
| *H1718* | response regulator transcription factor | 0.90 |
| *H1819* | response regulator receiver domain-containing protein | 0.07 |
| *H1832* | response regulator | -0.76 |
| *H1833* | response regulator | 0.49 |
| *H2031* | response regulator | -1.41 |
| *H2216* | response regulator | 1.48 |
| *H2360* | response regulator | -2.85 |
| *H2548* | response regulator transcription factor | 0.49 |
| *H2834* | response regulator | 1.34 |
| *H2835* | response regulator | 1.34 |
| *H2862* | response regulator | 1.20 |
| *H2868* | response regulator | -1.18 |
| *H2971* | response regulator receiver protein | 2.83 |
| *H2986* | response regulator transcription factor | 0.18 |
| *H3073* | response regulator transcription factor | 0.29 |
| *H3091* | response regulator transcription factor | 0.46 |
| *H3125* | response regulator transcription factor | -0.52 |
| *H3128* | response regulator transcription factor (RpaB) | 0.84 |
| *H3221* | PleD family two-component system response regulator | 0.33 |
| *H3335* | response regulator | 1.31 |
| *H3341* | response regulator | 0.06 |
| *H3527* | response regulator | 0.13 |
| *H3713* | response regulator transcription factor | 0.48 |
| *H3776* | response regulator transcription factor | -1.06 |
| *H3795* | response regulator transcription factor | -1.01 |

Table S3. Quantities of total soluble protein in *Halothece*.

|  | Total soluble protein (μg/mg fresh weight) | | | | | | |
| --- | --- | --- | --- | --- | --- | --- | --- |
| Hours in LL | 4 | 10 | 16 | 22 | 28 | 34 | Average ± SE |
| 0.5 M NaCl | 54.5 | 55.2 | 50.8 | 54.0 | 55.2 | 60.7 | 55.1 ± 1.3 |
| 2.5 M NaCl | 42.2 | 44.5 | 43.2 | 48.7 | 46.3 | 46.2 | 45.2 ± 1.0 |

The total soluble protein extracted for Western blot analysis was quantified using the Bradford method. The values for each LL time and averaged values of LL4-34 are shown. The values for each LL time were averaged values of two independent experiments.

Table S4. Primers used for RT-PCR.

| gene | Sequence (5’ to 3’) | Ref. | Cycle number |
| --- | --- | --- | --- |
| *H3510* | Up: GCGAGAATCCCATTGAAG  Low: CCTTCATCCAGAATCAAG | This study | 25 |
| *H3511* | Up: ACTACGACAGTGGTAGTG  Low: GGACAATTATCGGTTTGC | This study | 23 |
| *H1072* | Up: GTCTCCTGATCCAGAAGGTC  Low: ACACATTTCATCGAGGGTAC | This study | 25 |
| *H1590* | Up: GGATCCAATGACGAAAACAACCTCTGC  Low: TGAGGATCGGTTTCCACAAG | ^6^ | 27 |
| *H1078* | Up: GGATCCAATGACGATCACTAACGATAAAC  Low: ATGCAGAATAGCCCGTAAAC | ^6^ | 27 |
| *H1077* | Up: GGATCCAATGCTTCTATTTGTCCGAGG  Low: ATAGTAACTAGAAACGGGAC | ^6^ | 27 |
| *H1076* | Up: GGATCCAATGTCTTTATTACGAGTTTTAC  Low: AAGCCTGTCTGTAACGCTGC | ^6^ | 27 |

**Supplemental Information References**

1 Kageyama, H. *et al.* An alkaline phosphatase/phosphodiesterase, PhoD, induced by salt stress and secreted out of the cells of Aphanothece halophytica, a halotolerant cyanobacterium. *Appl Environ Microbiol* **77**, 5178-5183, doi:10.1128/AEM.00667-11 (2011).

2 Patipong, T., Hibino, T., Waditee-Sirisattha, R. & Kageyama, H. Induction of antioxidative activity and antioxidant molecules in the halotolerant Cyanobacterium *Halothece* sp. PCC7418 by Temperature Shift. *Natural Product Communications* **14**, 1-6, doi:10.1177/1934578x19865680 (2019).

3 Patipong, T. *et al.* A class I fructose-1,6-bisphosphate aldolase is associated with salt stress tolerance in a halotolerant cyanobacterium *Halothece* sp. PCC 7418. *Arch Biochem Biophys* **672**, 108059, doi:10.1016/j.abb.2019.07.024 (2019).

4 Kanesaki, Y., Suzuki, I., Allakhverdiev, S. I., Mikami, K. & Murata, N. Salt stress and hyperosmotic stress regulate the expression of different sets of genes in *Synechocystis* sp. PCC 6803. *Biochem Biophys Res Commun* **290**, 339-348, doi:10.1006/bbrc.2001.6201 (2002).

5 Al-Hosani, S., Oudah, M. M., Henschel, A. & Yousef, L. F. Global transcriptome analysis of salt acclimated *Prochlorococcus* AS9601. *Microbiol Res* **176**, 21-28, doi:10.1016/j.micres.2015.04.006 (2015).

6 Waditee-Sirisattha, R., Kageyama, H., Sopun, W., Tanaka, Y. & Takabe, T. Identification and upregulation of biosynthetic genes required for accumulation of Mycosporine-2-glycine under salt stress conditions in the halotolerant cyanobacterium *Aphanothece halophytica*. *Appl Environ Microbiol* **80**, 1763-1769, doi:10.1128/AEM.03729-13 (2014).
